# Coronary Artery Disease Associated Transcription Factor TCF21 Regulates Smooth Muscle Precursor Cells That Contribute to the Fibrous Cap

**DOI:** 10.1371/journal.pgen.1005155

**Published:** 2015-05-28

**Authors:** Sylvia T. Nurnberg, Karen Cheng, Azad Raiesdana, Ramendra Kundu, Clint L. Miller, Juyong B. Kim, Komal Arora, Ivan Carcamo-Oribe, Yiqin Xiong, Nikhil Tellakula, Vivek Nanda, Nikitha Murthy, William A. Boisvert, Ulf Hedin, Ljubica Perisic, Silvia Aldi, Lars Maegdefessel, Milos Pjanic, Gary K. Owens, Michelle D. Tallquist, Thomas Quertermous

**Affiliations:** 1 Department of Medicine, Cardiovascular Research Institute, Stanford University School of Medicine, Stanford, California, United States of America; 2 Department of Medicine, University of Hawaii, Honolulu, Hawaii, United States of America; 3 Department of Molecular Medicine and Surgery, Karolinska Institute, Stockholm, Sweden; 4 Department of Medicine, Karolinska Institute, Stockholm, Sweden; 5 Department of Molecular Physiology and Biological Physics, University of Virginia School of Medicine, Charlottesville, Virginia, United States of America; University of Oxford, UNITED KINGDOM

## Abstract

Recent genome wide association studies have identified a number of genes that contribute to the risk for coronary heart disease. One such gene, *TCF21*, encodes a basic-helix-loop-helix transcription factor believed to serve a critical role in the development of epicardial progenitor cells that give rise to coronary artery smooth muscle cells (SMC) and cardiac fibroblasts. Using reporter gene and immunolocalization studies with mouse and human tissues we have found that vascular *TCF21* expression in the adult is restricted primarily to adventitial cells associated with coronary arteries and also medial SMC in the proximal aorta of mouse. Genome wide RNA-Seq studies in human coronary artery SMC (HCASMC) with siRNA knockdown found a number of putative *TCF21* downstream pathways identified by enrichment of terms related to CAD, including “vascular disease,” “disorder of artery,” and “occlusion of artery,” as well as disease-related cellular functions including “cellular movement” and “cellular growth and proliferation.” In vitro studies in HCASMC demonstrated that *TCF21* expression promotes proliferation and migration and inhibits SMC lineage marker expression. Detailed *in situ* expression studies with reporter gene and lineage tracing revealed that vascular wall cells expressing *Tcf21* before disease initiation migrate into vascular lesions of *ApoE^-/-^* and *Ldlr^-/-^* mice. While *Tcf21* lineage traced cells are distributed throughout the early lesions, in mature lesions they contribute to the formation of a subcapsular layer of cells, and others become associated with the fibrous cap. The lineage traced fibrous cap cells activate expression of SMC markers and growth factor receptor genes. Taken together, these data suggest that *TCF21* may have a role regulating the differentiation state of SMC precursor cells that migrate into vascular lesions and contribute to the fibrous cap and more broadly, in view of the association of this gene with human CAD, provide evidence that these processes may be a mechanism for CAD risk attributable to the vascular wall.

## Author Summary

Coronary artery disease (CAD) is responsible for the majority of deaths in the Western world, and is due in part to environmental and metabolic factors. However, half of the risk for developing heart disease is genetically predetermined. Genome-wide association studies in human populations have identified over 100 sites in the genome that appear to be associated with CAD, however, the mechanisms by which variation in these regions are responsible for predisposition to CAD remain largely unknown. We have begun to study a gene that contributes to CAD risk, the *TCF21* gene. Through genomic studies we show that this gene is involved in processes related to alterations in vascular gene expression, and in particular those related to the smooth muscle cell biology. With cell culture models, we show that *TCF21* regulates the differentiation state of this cell type, which is believed critical for vascular disease. Using mouse genetic models of atherosclerotic vascular disease we provide evidence that this gene is expressed in precursor cells that migrate into the disease lesions and contribute to the formation of the fibrous cap that is believed to stabilize these lesions and prevent heart attacks.

## Introduction

Atherosclerotic coronary heart disease (CAD) continues to be the dominant medical problem in the Western world and is growing in minority groups in this country and in developing populations [1–4]. Despite extensive investigation of epidemiological and cellular features of the disease, there are still fundamental questions related to the mechanism of etiology that remain to be answered. For instance, the resident SMC that constitute the majority of the native vessel wall undergo dramatic phenotypic changes in the disease setting, with the loss of SMC gene expression in the medial vessel layer and appearance of a new layer of SMC marker expressing cells in a “fibrous cap” that covers the lipid core of the plaque. While the risk of plaque rupture appears to be inversely correlated with the number of SMC-like cells in the fibrous cap, there is very little understanding of their in vivo origin, and the molecular pathways that regulate their expansion and terminal phenotype determination [5]

Significant new insights into the fundamental cellular processes that drive CAD and other complex human diseases have recently been achieved through genome wide association (GWA) studies employing large cohorts of patients and healthy controls. These studies have provided the first incontrovertible identification of genes and disease pathways that affect risk for many complex human diseases (www.genome.gov/gwastudies), including CAD. A recent GWA meta-analysis has identified 46 loci that associate with CAD at the genome-wide significance level of 5 x 10^-8^, and another 104 independent variants that associate at a false discovery rate less than 0.05 [6]. Of these CAD associated loci, more than two-thirds act independently of traditional risk factors, and are likely to modulate risk of CAD through regulation of cellular processes in the blood vessel wall. Further study of this latter group of genes is expected to provide insights into novel atherosclerosis pathways.

One CAD associated gene that has not been linked to known environmental or metabolic risk factors is the basic-helix-loop-helix transcription factor *TCF21* (capsulin, *POD1*, epicardin) [6–8]. This gene was initially cloned in four independent laboratories and shown to mark expression of mesodermal cells that give rise to kidney, lung, spleen, gonads, and a restricted group of cranial muscle cells [9–16]. Recent studies have shown that *Tcf21* regulates fundamental cell fate decisions in the developing epicardium, serving as a determining factor for divergence between coronary vascular smooth muscle cell and cardiac fibroblast lineages [17,18]. In this setting, *Tcf21* is downregulated in cells that are fated to become differentiated coronary SMC and has sustained expression in cells that become interstitial and adventitial fibroblasts [18]. However, its role in molecular and cellular processes related to CAD has not been investigated.

To examine the possible role of *TCF21* in coronary artery disease, we assessed its expression pattern in normal and diseased tissues, investigated its role in fundamental cellular processes, and employed next generation sequencing based methods in SMC to identify genes and pathways regulated by *TCF21* at the transcriptional level. We have also identified the temporal and cell-specific expression of *Tcf21* in reporter gene and lineage tracing mouse models to better understand how this gene might regulate vascular wall cellular processes in the setting of atherosclerotic disease.

## Results

### TCF21 is expressed in human SMC and other mesodermal lineages in vitro and in human and mouse coronary arteries

To better understand the expression pattern of *TCF21* in adult tissues, we studied expression in a number of adult human cells by quantitative real-time polymerase chain reaction (qPCR) (S1A Fig). *TCF21* was expressed in human coronary artery smooth muscle cells (HCASMC), but not aortic smooth muscle cells (HAoSMC) or endothelial cells isolated from coronary (HCAEC) or aortic vessels (HAoEC). Because of the reported similarity between pericytes, which have been developmentally linked to *Tcf21* [10], and mesenchymal stem cells (MSC) [19], we investigated expression in MSC derived from bone marrow (bmMSC), embryonic-stem cells (eMSC) and induced pluripotent stem cells (iMSC). Results showed that all of these cells expressed *TCF21*. Cardiac fibroblasts (HCF) and adventitial aortic fibroblasts (HAoAF) were also found to express *TCF21*.

Immunocytochemistry of cultured HCASMC revealed nuclear expression of TCF21 in cells that were also expressing the SMC marker ACTA2, although a number of prominent cells expressing high levels of ACTA2 appeared to have low-level expression of TCF21 (S1B Fig). *In situ* hybridization of mouse and human coronary artery specimens with species-specific cRNA probes revealed prominent labeling of adventitial cells with no staining of medial SMC or intimal endothelial cells (S1C Fig).

In preparation for studies in mouse genetic disease models, we investigated *Tcf21* expression in normal adult mice with Xgal staining of tissues containing a *lacZ* reporter gene integrated into the murine *Tcf21* locus, and compared this staining with that seen in C57BL/6 mice [12,20]. As with in situ hybridization studies in mouse and human tissues, results showed staining of cells in the adventitia of both the coronary arteries and aorta, with primary localization in those adventitial cells juxtaposed to the vascular media (S1D Fig). Unique to the mouse, there was a patchy distribution of *lacZ* expressing cells in the proximal aortic wall (S1E Fig). When β-galactosidase activity was pseudocolored green and merged with the Acta2 immunoreactivity (red) there appeared to be minimal overlap in either the coronary arteries or aorta.

### Genomic studies identify disease and developmental pathways downstream of TCF21

To provide functional insights into the role of *TCF21* in adult SMC in the disease setting, we employed whole transcriptome RNA sequencing (RNA-Seq) studies on cells transfected with either non-silencing control siRNA (siCTRL) or silencing *TCF21* siRNA (si*TCF21*). Quantitative RT-PCR of HCASMC transfected with si*TCF21* compared to siCTRL showed a significant decrease in mRNA levels for *TCF21* (0.99±0.02 control vs. 0.25±0.02 si*TCF21*, *P*<0.0001) (S2A Fig), and quantitation of western blots of protein extracts showed a similar reduction of TCF21 protein levels to 26% of baseline (S2B Fig).

We employed two commonly used algorithms, DESeq and edgeR, to identify genes that were differentially expressed (FDR ≤ 0.05) between the HCASMC treated with si*TCF21* or siCTRL reagents [21,22]. To eliminate outliers that might be identified by each analysis, we intersected the 466 genes obtained with DESeq and 430 genes found with edgeR to obtain 380 genes that were common to the two approaches [23]. The intersected genelist was mapped to the Ingenuity IPA Knowledge Base, generating algorithmically computed pathways or networks that were then investigated for over-representation in disease and biological function categories. “Connective Tissue Development and Function, Tissue Morphology, Cardiovascular Disease” was identified as the top network. “Cardiovascular Disease” was ranked third in the disease category, with 20 subcategories including “Vascular disease” (Q = 3.26E-17, right-tailed Fisher’s Exact Test, Benjamini-Hochberg corrected), “Disorder of artery” (Q = 4.04E-12), and “Occlusion of artery” (Q = 2.03E-14) as the top three functional groups (Tables 1 and S1). “Cardiovascular System Development and Function” (Q = 1.88E-16- 5.30E-04) was the most highly ranked in the “Physiological System Development and Function” category, with a number of subcategories that were related to embryonic development, including “Development of the cardiovascular system” (Q = 1.88E-16), “Angiogenesis” (Q = 4.58E-14), and “Development of blood vessel” (Q = 4.58E-14) being the top three functional groups (Tables 1 and S2). Among the Molecular and Cellular Functions category of traits, the top three functional groups were “Cellular movement” (Q = 4.23E-23), followed by “Cellular growth and proliferation” (Q = 4.23E-23), and “Cell death and survival” (Q = 1.06E-18) (Tables 1 and S3).

**Table 1:** Functional annotation derived from analysis of differentially expressed genes in si*TCF21* RNA-Seq experiments.

CARDIOVASCULAR DISEASE	
***Functional annotation***	***Q-Value***
Vascular disease	3.26E-17
Disorder of artery	8.47E-17
Occlusion of artery	4.04E-12
Aneurysm	2.99E-09
Vascular lesion	6.68E-08
Peripheral arterial occlusive disease	1.23E-07
Aortic aneurysm	2.53E-07
Aortic disorder	7.58E-07
Arteriosclerosis	2.15E-06
Atherosclerosis	4.63E-06
Arterial dissection	2.31E-05
Cardiomyopathy	3.34E-05
Advanced stage peripheral arterial occlusive disease	4.35E-05
Mid-stage peripheral arterial occlusive disease	7.57E-05
Infarction	8.59E-05
**CARDIOVASCULAR SYSTEM DVLP & FUNCTION**	
***Functional annotation***	***Q-Value***
Development of cardiovascular system	1.88E-16
Angiogenesis	4.58E-14
Development of blood vessel	4.58E-14
Morphology of cardiovascular system	2.20E-13
Vasculogenesis	6.32E-13
Abnormal morphology of cardiovascular system	8.11E-11
Cell movement of endothelial cells	4.37E-10
Migration of endothelial cells	5.11E-09
Adhesion of endothelial cell lines	2.03E-08
Endothelial cell development	3.82E-08
Neovascularization	1.67E-07
Angiogenesis of tumor	4.63E-07
Angiogenesis of extraembryonic tissue	5.39E-07
Proliferation of endothelial cells	1.68E-08
Morphology of blood vessel	1.71E-06
Abnormal morphology of blood vessel	2.09E-06
**MOLECULAR AND CELLULAR FUNCTIONS**	
***Functional annotation***	***Q-Value***
Cellular movement	4.23E-23
Cellular growth and proliferation	4.23E-23
Cell death and survival	1.06E-18
Cellular development	2.76E-14
Cellular function and maintenance	2.10E-09

**Fig 1:**
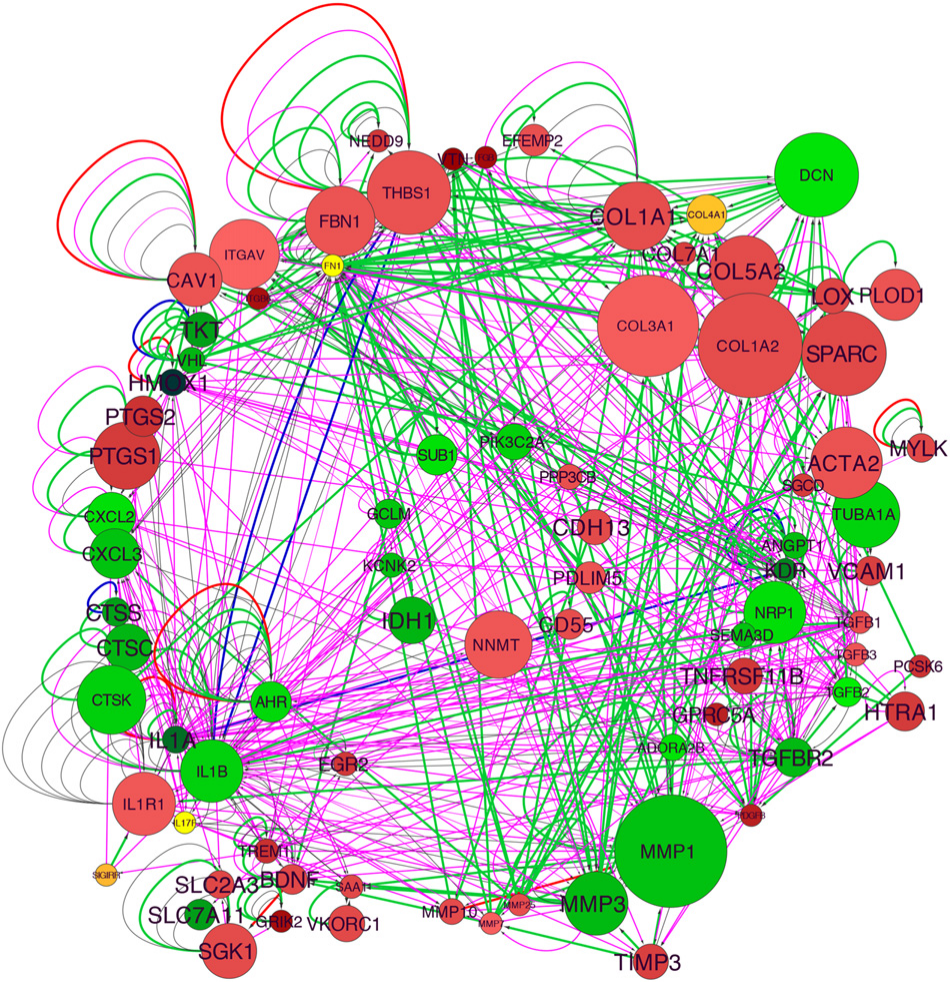
TCF21 Vascular Disease Network built with differential gene expression data from si*TCF21* RNA-Seq studies in HCASMC. Interaction of the network nodes identified through enrichment of differentially expressed genes in functionally annotated categories was visualized with Cytoscape. Node color was mapped to log fold change with green representing genes that are downregulated along with *TCF21* and red representing genes that are upregulated, node size was mapped to absolute expression value in control cells, and font size to enrichment *Q*-value. Edges contain arrows to indicate the direction of interaction and are colored to distinguish types of interactions. Green edges represent functional interaction (protein-protein binding, protein modification, molecular cleavage, phosphorylation, and protein-DNA interactions); magenta edges represent gene expression (expression and transcription) relationships; red edges represent activation; and blue edges inhibition.

Taking the first subcategory in the “Cardiovascular Disease” category, “Vascular disease,” we used well-curated molecular interactions in the IPA Knowledge Base to build a gene network. The initial group included 73 genes, and 18 additional genes were added with the “build” function in IPA, this group functionally related with the “connect” function, and with elimination of non-connected nodes resulted in a final *TCF21* Vascular Disease Network containing 83 genes (Fig 1). Also, content was added to the network with annotation from Gene Ontology classifications (S3 Fig). One of the strongest signals in the network was the upregulation of matrix components with *TCF21* knockdown, including a large number of collagens, *SPARC*, *LOX*, vitronectin (*VTN*), and components of the matrix that are known to affect vascular cell functions, including thrombospondin 1 (*THBS1*) and fibrillin 1 (*FBN1*), as well as vascular cell surface proteins that interact with the matrix including alpha V integrin (*ITGAV*). Although the matrix degrading enzymes *MMP7*, *MMP10* and *MMP25* are upregulated with *TCF21* knockdown, the downregulation of the much more highly expressed *MMP1* and *MMP3* combined with the upregulation of MMP inhibitor *TIMP3* are consistent with an anti-matrix overall profile of a mobile cell. Also upregulated are SMC markers such as smooth muscle actin (*ACTA2*) and myosin light chain kinase (*MYLK*). Among the genes downregulated with *TCF21* knockdown are those involved in vascular development, including neuropilin 1 (*NRP1*), VEGF receptors II (*KDR*), angiopoietin 1 (*ANGPT1*), TGFβ receptor 2 (*TGFBR2*), and semaphorin 3D (*SEMA3D*), and cytokines and chemokines such as *IL1A*, *IL1B*, *CXCL2* and *CXCL3*. Taken together these data suggest that *TCF21* positive cells express low levels of many matrix proteins while expressing high levels of matrix degrading enzymes, consistent with a pro-migratory phenotype, express low levels of SMC lineage markers, and are highly pro-angiogenic and pro-inflammatory, a phenotype quite different from vascular wall SMC.

### TCF21 directly regulates cell fate decisions in cultured SMC

To investigate a functional role for *TCF21* in fundamental cell fate decisions in vascular SMC, we employed lentiviral shRNA as well as siRNA mediated knockdown and lentiviral over-expression studies. Control lentiviral vectors (pWPI) and lentiviral over-expression vectors (pWPI-*TCF21*), and control (pLVTHM) and lentiviral shRNA mediated knockdown vectors (pLVTHM-sh*TCF21*) were used to transduce primary cultured HCASMC. pWPI-*TCF21* increased *TCF21* mRNA levels (1.0±0.04 pWPI vs. 32.5±0.02 pWPI-*TCF21*, P<0.0001), and pLVTHM-sh*TCF21* decreased expression (1.0±0.06 pLVTHM vs. 0.34±0.04 pLVTHM-sh*TCF21* 2, P<0.001) (S4A Fig). Western blot of protein extracts from HCASMC that were transduced with over-expression and knockdown lentiviruses showed a 4.5-fold increase, and reduction of TCF21 protein levels to 8% (sh*TCF21* 1, sh*TCF21* 2) of baseline respectively (S4B Fig).

**Fig 2:**
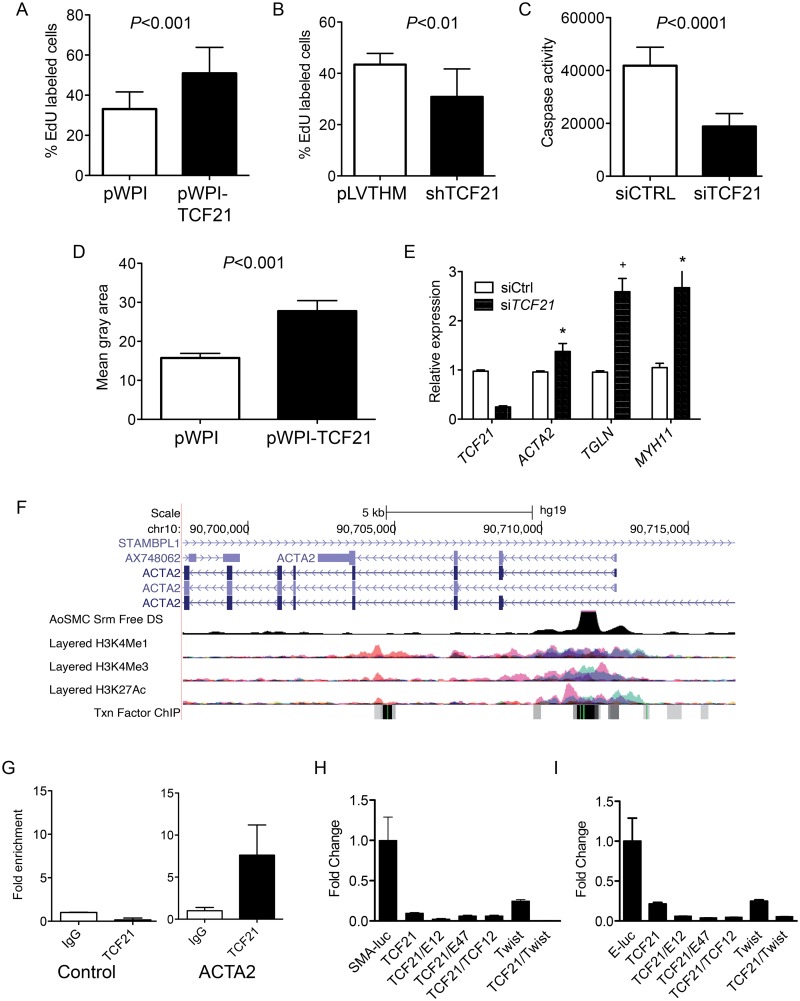
*TCF21* regulates basic cellular functions in vascular smooth muscle cells *in vitro*. A) HCASMC transduced with *TCF21* overexpressing lentivirus (pWPI-*TCF21*) or empty lentivirus (pWPI empty) were labeled with the thymidine analogue 5-ethynyl-2′-deoxyuridine (EdU), which was visualized with a fluorescent azide, allowing identification and quantification of proliferating cells with immunofluorescence microscopy, reported here as a percent with baseline being all DAPI positive cells. HCASMC showed an increase in the percentage of *TCF21* overexpressing cells compared to DAPI stained cells (33.1% ± 2.3 control vs. 51% ± 4.1 overexpressing cells, *P*<0.001). B) HCASMC transduced with knockdown lentiviruses showed a decreased percentage of dividing cells (43.4% ± 4.4 control vs. 30.8% ± 2.4 knockdown cells, *P*<0.01). C) s*iTCF21* produced a decrease in apoptosis in HCASMC as measured by a caspase activity assay (41,824±1872 vs. 18,837±1302, *P*<0.0001). D) *TCF21* regulation of HCASMC migration was evaluated with a gap closure assay. *TCF21* overexpressing cells transduced with pWPI-*TCF21* lentivirus covered a significantly larger surface area after 12 hours of study compared to cells transduced with the empty pWPI vector (27.8 ± 2.7 vs. 15.8 ± 1.16, *P*<0.001). E) HCASMC treated with si*TCF21* compared to siCTRL showed significantly increased expression of *ACTA2*, *TAGLN*, and *MYH11* SMC marker genes (*P*<0.05/*P*<0.01/*P*<0.05 respectively). F) The *ACTA2* locus as visualized on the University of California Santa Cruz genome browser. Data provided here reveals evidence for a likely enhancer region in the first intron, as indicated by DNase hypersensitivity measured in human aortic SMC, and histone modification data showing enrichment of H3K27Ac and H3K4me1 at this same site, as well as clustering of a number of transcription factor binding sites. G) Chromatin immunoprecipitation for *TCF21* binding to the enhancer region of the *ACTA2* locus by ChIP-qPCR (*P*<0.05). H) Dual luciferase assays in rat aortic smooth muscle cells with a reporter construct containing the human *ACTA2* promoter and first intron (SMA-luc). A *TCF21* expression construct was transfected with human *TCF3 (E12)*, *TCF3 (E47)*, *TCF12*, and *Twist1* murine expression vectors, showing specific suppression of transcription of the SMA-luc reporter (1.0 ± 0.01 vs. 0.1 ± 0.009, *P*<0.01 for *TCF21* alone). I) Similar dual luciferase assays using a 3 E-box containing minimal promoter construct (E-luc) based on the nucleotide sequence of the first intron (n = 3, 3 replicates), again showing *TCF21* mediated suppression of transcription (1.0 ± 0.29 vs. 0.21 ± 0.02, *P*<0.01 for *TCF21* alone).

To assess the effect of *TCF21* on rate of cell division in vitro, proliferation rates of pools of stably transduced and non-transduced cells were measured over time, where increased or decreased proliferation rates in transduced cells would lead to a change in percentages of this GFP positive cell population as compared to the non-transduced, GFP negative population. Flow cytometry analysis of stable overexpression of *TCF21* in HCASMC showed an increase in *TCF21* overexpressing cells from 48 to 82 percent of the culture within 28 days (S5A Fig). In the same time period the percentage of cells with stable knockdown of *TCF21* decreased from 53 to 16 for construct 1 (sh*TCF21* 1 vs. empty control virus pLVTHM *P*<0.0001) and from 59 to 2 percent for construct 2 (sh*TCF21* 2 vs. empty control virus pLVTHM *P*<0.0001) (S5B Fig). The percentage of GFP positive cells did not change when transduced with the empty vector control construct. To provide additional support for these findings we conducted EdU (5-ethynyl-2’-deoxyuridine) labeling proliferation assays. HCASMC transduced with the control, overexpression or knockdown lentiviruses were treated with EdU and imaged for nuclear fluorescence. These data showed an increase in the percentage of *TCF21* overexpressing cells compared to DAPI stained cells (33.1% ± 2.3 control vs. 51% ± 4.1 overexpressing cells, *P*<0.001), and significant decreased percentage of dividing cells transduced with knockdown lentiviruses (43.4% ± 4.4 control vs. 30.8% ± 2.4 knockdown cells, *P*<0.01) (Fig 2A and 2B). These data suggest that *TCF21* positively affects SMC proliferation.

We also evaluated the effect of *TCF21* on apoptosis and migration in cultured HCASMC. Apoptosis was evaluated in HCASMC transfected with siControl or si*TCF21* RNAs and serum starved for 48 hrs. By caspase assay there was a significant decrease in apoptosis of cells transfected with the si*TCF21* reagent (41,824±1872 vs. 18,837±1302 for siCTRL compared to si*TCF21*, *P*<0.0001) (Fig 2C). For migration, HCASMC transduced with *TCF21* overexpression lentivirus were evaluated with a gap closure assay. Here, *TCF21* overexpressing cells covered a significantly larger surface area after 12 hours of incubation (27.8 ± 2.7 vs. 15.8 ± 1.16 control cells, *P*<0.001) (Fig 2D). With an average doubling time of 71 hours for *TCF21* overexpressing HCASMC this effect could not be due to the pro-proliferative effect of *TCF21*.

Given that *TCF21* is downregulated in epicardial cells as they differentiate into coronary SMC, and a number of SMC lineage marker genes were upregulated in the siTCF21 RNA-Seq study, including *ACTA2* and *MYLK*, we specifically investigated the expression of SMC marker genes in additional siRNA knockdown experiments. These studies showed that *ACTA2*, *TAGLN* and *MYH11* transcript levels were significantly upregulated after 70% *TCF21* knockdown (0.96 ± 0.023 vs. 1.38 ± 0.16, *P*<0.05, 1.0 ± 0.03 vs. 2.59 ± 0.27, *P*<0.01, and 1.05 ± 0.09 vs. 2.67 ± 0.51, *P*<0.05, respectively) (Fig 2E), suggesting their direct suppression by TCF21 at the transcriptional level.

To investigate whether TCF21 directly binds and regulates SMC marker genes, a series of studies were conducted with the *ACTA2* gene as a representative SMC marker. It is well known that the regulatory regions of the *ACTA2* gene are localized in the upstream promoter and first intronic regions of the gene [24], and this information in conjunction with available ENCODE histone modification data, ENCODE DNase hypersensitivity data from human aortic smooth muscle cells, and localization of E-box binding motif sequences provided the approach for choosing a reporter construct for these studies (Fig 2F). Chromatin immunoprecipitation was employed to verify the in vivo binding of TCF21 to a region of the first intron enhancer identified through this analysis (Fig 2G). The luciferase reporter gene construct employed for these studies contained the human *ACTA2* promoter and first intron. Obligate heterodimer partners of TCF21, TCF3 and TCF12, were included in these experiments to facilitate transcriptional activation [25]. Also, based on the high degree of conservation of sequence in the bHLH domain between TCF21 and Twist, and the similar inhibitory developmental actions of this related transcription factor, an expression construct for Twist was included to investigate possible interactions with TCF21 in the context of *ACTA2* reporter gene expression [26]. When this reporter was transfected into rat aortic smooth muscle cells (RASMC) in conjunction with a *TCF21* expression construct, it was found to decrease expression (1.0 ± 0.01 vs. 0.1 ± 0.009, *P*<0.05), and even greater reductions were observed when the *TCF21* expression vector was combined with another vector expressing one of the class II bHLH factors TCF3 (protein isoforms E12 or E47) (Fig 2H). *TCF21* was also shown to inhibit expression of a minimal luciferase reporter containing 3 E-box sequences based on the known TCF12 motif (CAGCTG) in conjunction with a minimal promoter. There was significant repression with *TCF21* expression alone (1.0 ± 0.29 vs. 0.21 ± 0.02, *P*<0.05), and additional reductions were noted when bHLH class II factors were included (Fig 2I). For both constructs, the developmental factor Twist produced a modest increase in TCF21 transrepression.

### TCF21 expressing cells are found in atherosclerotic lesions and associate with the fibrous cap

**Fig 3:**
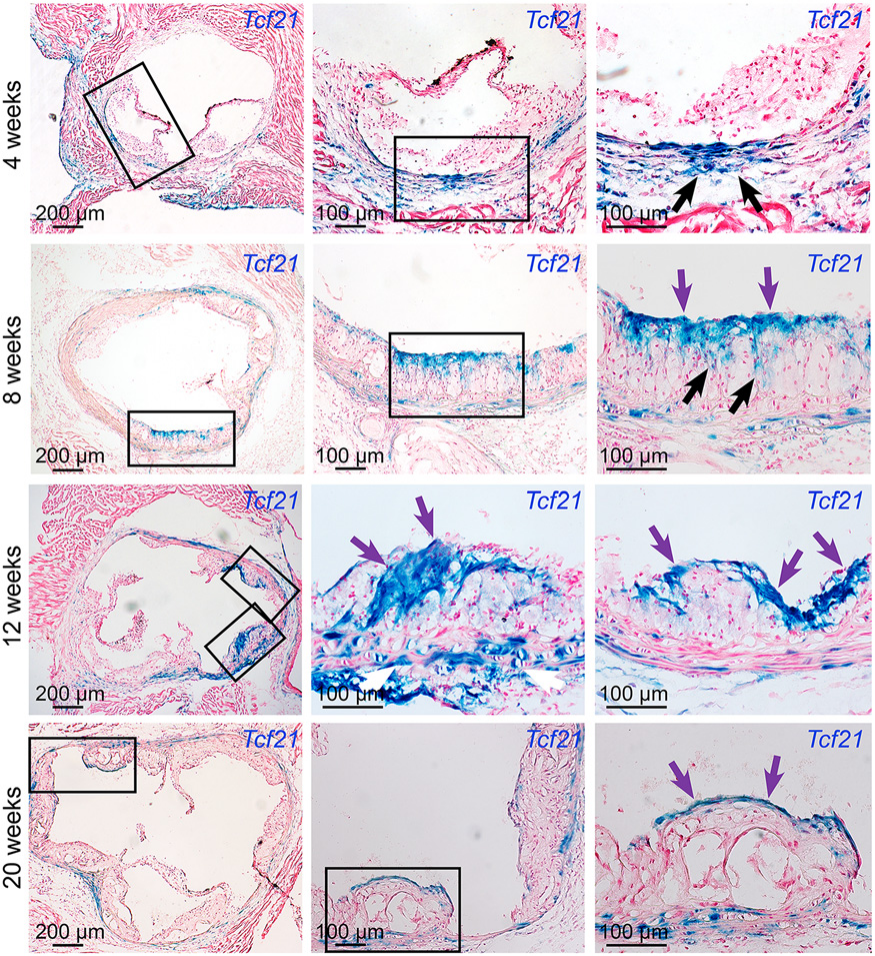
*Tcf21* expressing cells are found in atherosclerotic lesions. *Tcf21* ^*lacZ/+*^, *ApoE* ^*-/-*^ mice were fed HFD from 4 weeks of age for 4, 8, 12, or 20 weeks, proximal aortic tissues were harvested, and *Tcf21* gene expression evaluated by Xgal staining to visualize β-galactosidase activity, and sections were counterstained with nuclear fast red to visualize disease lesion architecture. For each timepoint, boxes in low-power images at left indicate regions examined at high power in panels to the right. At 4 weeks of HFD there was no expression in the lesions although clusters of *lacZ* expressing cells identified in the media in regions below the disease lesions (arrows). At the 8-week timepoint cells with β-galactosidase activity were seen extending from the media to the luminal surface of the lesion (black arrows) in the vicinity of the forming fibrous cap (purple arrows). By 12 weeks of HFD there was extensive labeling of cells in lesions, with the appearance of β-galactosidase positive cells in the vicinity of the fibrous cap (purple arrows). Also, there was extensive staining of cells in areas of disrupted medial structure (white arrows). After 20 weeks of HFD, *Tcf21*-expressing cells had decreased in the lesions but formed a narrow band of cells associated with the fibrous cap (purple arrows).

**Fig 4:**
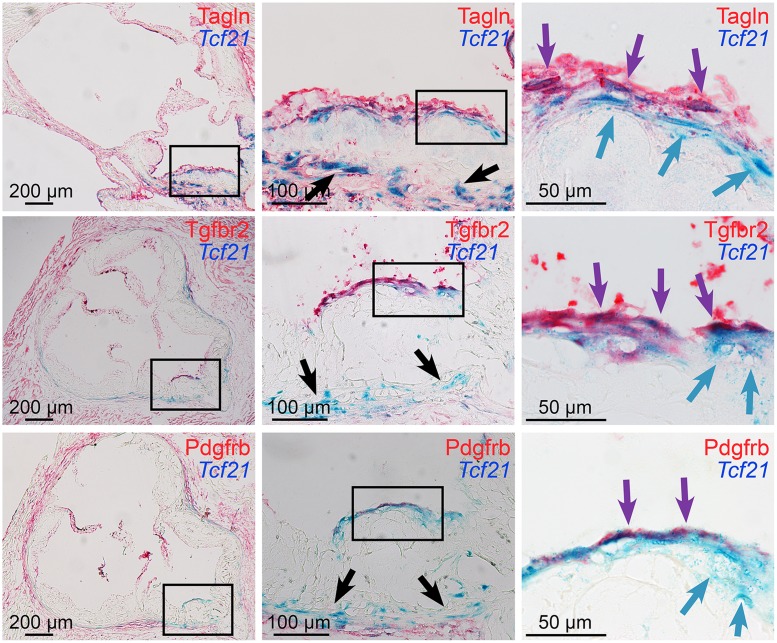
*Tcf21* expressing cells associate with the fibrous cap. Xgal stained lesions in *Tcf21* ^*lacZ/+*^, *ApoE* ^*-/-*^ mice fed HFD for 20 weeks were evaluated by immunohistochemistry for expression of fibrous cap markers. *Tcf21* reporter expressing cells (blue indicator) were identified in the media and adventitia (black arrows, middle panels), and in association with the fibrous cap (blue arrows, right panels). β-galactosidase negative cells in the luminal aspect of the fibrous cap stained positive for Tagln, as well as growth factor receptors Tgfbr2 and Pdgfrb (red indicator). In addition, in the region of the fibrous cap, cells were identified that showed staining for *Tcf21* expression as well as Tagln and growth factor receptors (purple arrows).

To investigate disease-related expression of *Tcf21* in the vascular wall, we performed time-course studies in *Tcf21* ^*lacZ/+*^, *ApoE* ^*-/-*^ hyperlipidemic atherosclerotic mice, and compared Xgal staining in these tissues with that observed in negative control *ApoE* ^*-/-*^ animals. After four weeks of high fat diet (HFD) there were no β-galactosidase expressing cells in the forming lesions, although there was apparent clustering of β-galactosidase positive cells on the luminal side of the media immediately below the areas where lesions had initiated growth (Fig 3). By eight weeks of HFD, lesions in most animals showed abundant β-galactosidase positive cells accumulating toward the luminal side of the atheromatous lesion, with streaks extending from media through the lesion toward the fibrous cap. β-galactosidase positive cells observed in the lesion appeared to be aligned orthogonal to the axis of orientation for cells in the media, suggestive of migratory behavior. By twelve weeks of HFD feeding there was extensive staining in lesions of most animals, with the β-galactosidase positive cells in the vicinity of the forming fibrous cap, or in a cap-like structure that was situated medial to and extended below the actual fibrous cap (Figs 3 and 4). This time interval from 4 to 12 weeks was associated with an apparent decrease in the number of medial cells expressing SMC markers and a disruption of the lamellar medial structure. *Tcf21* expressing cells in the media at 8 to 12 weeks were disorganized and had lost their characteristic lamellar structure. By 20 weeks, almost all of the *Tcf21* expressing cells in the lesions were associated with the fibrous cap or a subcapsular structure (Fig 3).

To determine whether results in the ApoE model were consistent with *TCF21* expression in the diseased human coronary vessel wall, immunostaining of human coronary artery tissues was performed with antibodies for TCF21 and ACTA2. In those vessels with only modest disease there was adventitial TCF21 staining with some staining of the minimal neointima, but no TCF21 staining in the media, which was positive for ACTA2 (S6A–S6C Fig). In vessels with significant disease there was robust TCF21 staining in the adventitia and neointima and some patchy staining in the fibrous cap, but very little staining in the media (S6D and S6E Fig). ACTA2 staining was prominent in the fibrous cap and considerably weaker staining in the media. These studies confirm what was observed in the mouse diseased vasculature.

To assign *TCF21* expressing cells to a known cellular lineage we evaluated combined *lacZ* expression with immunohistochemistry staining for well-known lineage markers for different vascular cell types. Specifically, we worked to identify co-staining for murine adventitial and vascular lesion lineage markers, focusing on *lacZ* reporter gene expression in the *ApoE* ^*-/-*^ mice. Xgal and immunohistochemistry staining was performed of adventitial and lesion areas with antibodies specific for endothelial cells (VWF), SMC (ACTA2), neuronal cells (TUBB3), adipocytes (PLIN1), fibroblasts (THY1), stem cells (nestin), macrophages (LAMP2), hematopoietic cells (PTPRC), and dendritic cells (Itgax) (S7 Fig). These studies failed to identify co-localization with known cellular lineage markers, and when combined with published negative data for pericyte markers argue that *Tcf21* is not expressed by a common terminally differentiated vascular cell type [17].

Further reporter gene and immunohistochemistry studies focused on the fibrous cap in lesions of mice fed HFD for 4 to 20 weeks. Studies with SMC marker Tagln again revealed that the majority of *Tcf21* expressing cells in the adventitia, media and plaque of diseased vessels did not express SMC markers, which were easily identified in association with medial and fibrous cap SMC (Fig 4). Although individual *Tcf21* expressing cells and those in subcapsular structures did not express SMC markers, there were *Tcf21* expressing cells identified immediately below or within the fibrous cap that appeared to also express Tagln. In addition to SMC lineage markers, we investigated expression of growth factor receptor Tgfbr2, which is involved in SMC development and structural vascular wall disease [27,28] and was identified as one of the differentially expressed genes in the TCF21 knockdown RNA-Seq studies, as well as Pdgfrb which has an important role in coronary SMC development and atherosclerotic disease [5,29]. Fibrous cap cells were shown to express growth factor receptors Tgfbr2 and Pdgfrb, and there appeared to be co-staining for *Tcf21* expression and growth factor expression by cells in the vicinity of the fibrous cap. Whether the observed co-staining identifies a unique transitional cell that expresses *Tcf21* and SMC marker genes, or is due simply to sustained enzymatic activity of the β-galactosidase protein after *Tcf21* expression is downregulated was not clear from these studies. However, these data suggest that *Tcf21* expressing cells can adopt an SMC phenotype and contribute to the fibrous cap.

### Tcf21 expressing cells give rise to smooth muscle cells in the fibrous cap of *ApoE* ^*-/-*^ mice

In the next series of experiments we sought to more clearly identify the possible origins and cellular fate of *Tcf21* expressing cells found in the vascular disease lesions. For these studies we employed a lineage tracing approach with an inducible Cre recombinase that provided permanent fluorophore marking of cells that expressed *Tcf21* at the time of activation of recombinase expression. We used a mouse line expressing a Cre recombinase protein fused to two mutant estrogen-receptor ligand-binding domains (MerCreMer) [30], under the control of the endogenous *Tcf21* locus (*Tcf21* ^*iCre/+*^). This line has been well characterized and used extensively to investigate the cellular fate of *Tcf21* expressing cells in the developing embryo [17,31]. For reporter alleles, we employed both a tandem dimer tomato (tdT) fluorescent reporter line (B6.Cg-*Gt(ROSA)26Sor* ^*tm14(CAGtdTomato)Hze*^/J) as well as a *lacZ* reporter line (B6.129S4-*Gt(ROSA)26Sor* ^*tm1Sor*^/J) that was tracked with histochemical β-galactosidase activity. *ApoE* ^*-/-*^ mice carrying the Cre and reporter alleles were administered tamoxifen before HFD and sacrificed after 16 weeks of the diet, or administered tamoxifen at 6–8 weeks and harvested after 8, 12 or 16 weeks of HFD before sacrifice.

**Fig 5:**
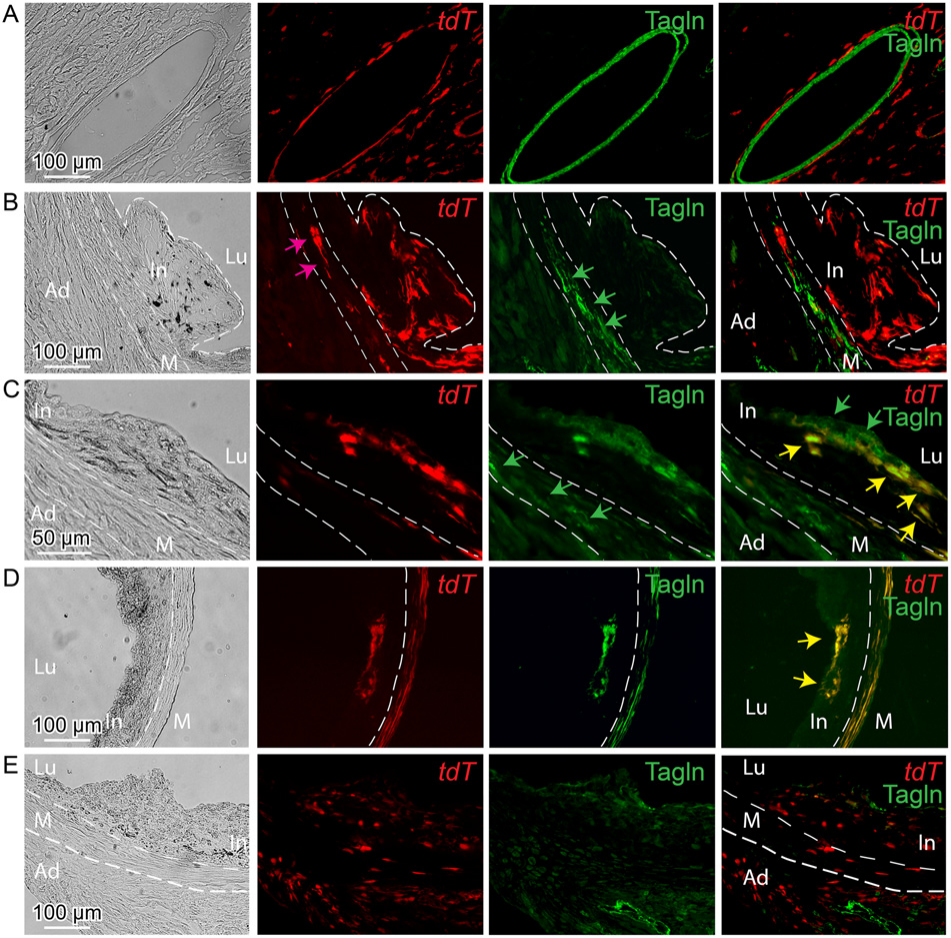
*Tcf21* expressing cells in *ApoE* ^*-/-*^ lesions give rise to smooth muscle cells in the fibrous cap. *Tcf21* ^*iCre/+*^, *ApoE* ^*-/-*^ mice were administered tamoxifen to activate expression of an inducible MerCreMer construct knocked into the *Tcf21* locus [31]. Cre mediated recombination of a tomato (tdT) reporter at the constitutively expressed *ROSA26* locus allowed lineage tracing of *Tcf21* expressing cells. Animals received tamoxifen at 6–8 weeks of HFD and tissues were harvested at 12 weeks of diet (A-D), or received tamoxifen 4–6 weeks of age prior to HFD and tissues harvested at 16 weeks (E). A) Intramyocardial coronary artery showing *Tcf21* expression (tdT) in adventitial cells surrounding the Tagln positive medial SMC. B) In an early lesion without a defined fibrous cap, tdT positive cells were seen throughout the lesion and in the media (pink arrow), with Tagln staining (green arrows) being restricted to cells in the media. C) In the Tagln and tdT merged imaging (right panel), lesion Tagln positive cells are identified in the fibrous cap (green arrows) and some of these cells co-stain for tdT fluorescence (yellow arrows), indicating that they previously expressed *Tcf21*. Tagln expressing cells in the media (green arrows, Tagln only imaging) do not colocalize with tdT staining in this location. D) Tagln positive cells in the fibrous cap were also positive for tdT fluorescence, suggesting that the identified SMC had expressed *Tcf21*. Also, co-staining of tdT and Tagln is noted in the media (yellow color). E) Animals received tamoxifen at 4 weeks of age prior to HFD and tissues were harvested at 12 weeks of diet. In an early lesion without a defined fibrous cap, tdT positive cells are seen throughout the adventitia, in the media and in the plaque. In, intima; M, media; Ad, adventitia; Lu, lumen.

Lineage tracing studies in *ApoE* ^*-/-*^ mice were primarily conducted in animals on HFD for 12 weeks, with tamoxifen being administered from 6 to 8 weeks of the diet. At baseline, *Tcf21* expression using this lineage tracing system was consistent with that seen for the *lacZ* knockin reporter mouse line, being restricted to the adventitial cells associated with coronary arteries, cardiac fibroblasts, and some aortic root cells (Fig 5A). Aortic root tissues from animals treated with HFD revealed tdT-expressing cells in the lesions. For early lesions without a defined fibrous cap, *Tcf21* lineage traced cells were distributed through the lesion and did not express the SMC marker Tagln (Fig 5B). In those lesions with a mature fibrous cap, *Tcf21* lineage traced cells were noted to contribute to this cap and many of these cells shown to express Tagln (Fig 5C and 5D). These data are consistent with the results described above for *Tcf21* reporter gene and SMC marker immunohistochemistry studies, and suggest that *Tcf21* expressing cells may give rise to SMC in the fibrous cap.

By activating recombinase activity before HFD feeding and lesion development it was possible to investigate the cellular origin of the *Tcf21* expressing cells identified in the vascular lesions of the *ApoE* ^*-/-*^ model. For mice that were administered tamoxifen before HFD, and then maintained on the diet for 12 weeks, lineage traced cells were commonly identified in lesions (Fig 5E). These data are thus consistent with their derivation from *Tcf21* expressing cells in the vascular wall adventitia or media, and suggest that *Tcf21* lineage traced cells were able to migrate into the forming lesions and contribute to lesion formation.

Finally, to verify the results obtained with the fluorescent tdT reporter, we conducted identical experiments with the Cre-activated *lacZ* reporter mouse. These studies also found that *Tcf21* lineage traced cells in the neointima did not express the SMC marker Tagln, whereas lineage traced cells contributing to the fibrous cap cells were found to express this SMC marker (S8 Fig).

### Lineage tracing in *Ldlr* ^*-/-*^ mice also shows recruitment of Tcf21 expressing cells that contribute to the fibrous cap

**Fig 6:**
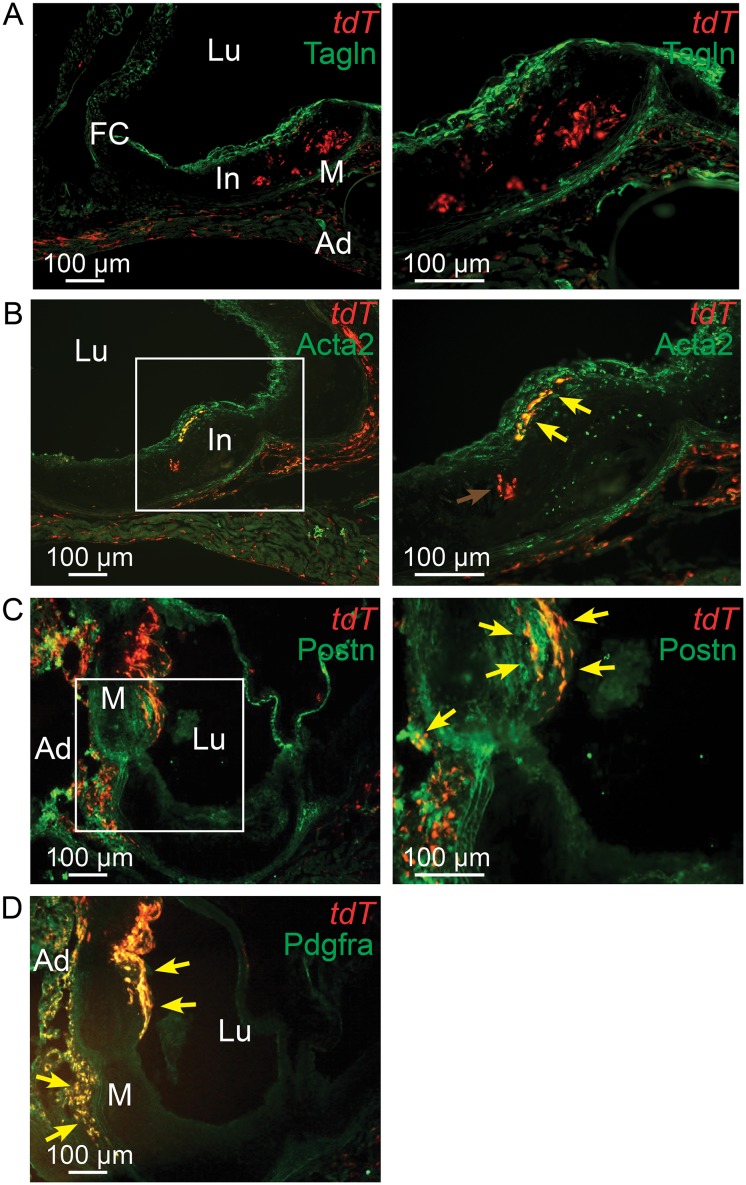
*Tcf21* lineage traced cells in *Ldlr* ^*-/-*^ lesions give rise to smooth muscle cells in the fibrous cap. *Tcf21* ^*iCre/+*^, *Ldlr* ^*-/-*^ mice were administered tamoxifen to activate expression of an inducible MerCreMer construct knocked into the *Tcf21* locus to produce recombination and thus expression of a floxed STOP tandem dimer tomato (tdT) reporter gene. Animals received tamoxifen chow at 3–5 weeks of age and were subsequently fed HFD for 16–20 weeks before sacrifice. A) tdT positive cells are seen throughout the lesion and in the adventitia with Tagln staining cells identified in the media and fibrous cap. B) A number of tdT lineage traced cells in the fibrous cap express the SMC marker Acta2 as shown by colocalization of fluorescent markers (yellow arrows). tdT positive cells within the lesion did not express Tagln, as shown by red color (red arrow). C) Some cells in the adventitia stain for periostin expression and colocalize with tdT staining in this location (yellow arrows). Staining for periostin and colocalization with *Tcf21* lineage cells was also observed in the fibrous cap and subcap (yellow arrows). D) Extensive colocalization was noted for Pdgfra antibody staining and tdT fluorescence in the adventitia and fibrous cap. In, intima; M, media; Ad, adventitia; FC, fibrous cap, Lu, lumen.

To generalize the findings obtained in the *ApoE* ^*-/-*^ model, we conducted lineage tracing studies in *Ldlr* ^*-/-*^ mice. Findings from these experiments also showed that activation of the reporter gene at 3 to 5 weeks of age, prior to HFD feeding for 16 to 20 weeks, marked cells that migrated into the atherosclerotic lesion (Fig 6A). As in the *ApoE* ^*-/-*^ model, some of the *Tcf21* traced cells activated expression of SMC markers Tagln and Acta2 and localized to the fibrous cap (Fig 6B). In addition, co-labeling with an antibody specific for the matrix protein periostin showed some general co-localization of *Tcf21* traced cells and periostin in the adventitia while there was extensive colocalization in the fibrous cap and co-staining in a subcap region located abluminal to the fibrous cap (Fig 6C). Also, *Tcf21* traced cells in the adventitia and fibrous cap showed extensive colocalization for Pdgfra expression (Fig 6D).

### The rate of cell division in vascular lesions is related to Tcf21 expression

**Fig 7:**
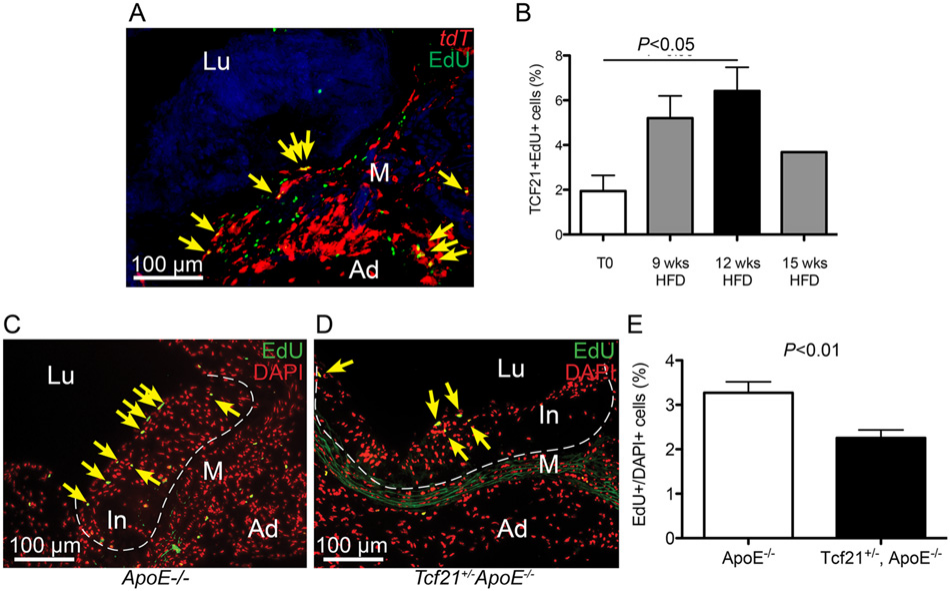
Rate of cell division in vascular lesions is related to Tcf21 expression. A) EdU staining was performed in *Tcf21* ^*iCre/+*^, *ROSA* ^*tdT/+*^, *Ldlr* ^*-/-*^ mice treated with tamoxifen and EdU and placed on HFD for 9, 12, or 15 weeks prior to tissue collection. Co-localization of *Tcf21* tdT fluorescence (red) and EdU fluorescent staining (green) allowed identification of the sdividing cells that were expressing *Tcf21* (yellow arrows, image from 12 week timepoint). B) The ratio of *Tcf21*+EdU+ cells compared to total *Tcf21*+ cells was significantly increased at 12 weeks of diet, consistent with a proliferative response in *Tcf21*+ cells. Comparison of percentage differences between 0 and 9 weeks, and from 9 to 12 weeks approached significance, *P* = 0.06; there was not a statistical difference between 12 and 15 weeks, *P* = 0.11. C, D) Rates of cell division in vascular lesions were compared between *Tcf21* ^*lacZ/+*^, *ApoE* ^*-/-*^ animals and *ApoE* ^*-/-*^ animals on HFD for 20 wks by quantifying the relative number of dividing cells identified by EdU fluorescence compared to the total number of cells identified by DAPI fluorescence. EdU fluorescence (green) was merged with red pseudocolored DAPI fluorescence, yellow arrows indicate yellow nuclei that are positive for both EdU and DAPI fluorescence. E) A statistically significant decrease in this percentage in *Tcf21* ^*lacZ/+*^, *ApoE* ^*-/-*^ animals compared to *ApoE* ^*-/-*^ animals suggested a correlation between *Tcf21* expression and rate of cell division in the vascular lesions. In, intima; M, media; Ad, adventitia; FC, fibrous cap, Lu, lumen.

To investigate whether *Tcf21* plays a role in regulating vascular cell proliferation in the disease setting *in vivo*, we used two different approaches, one employing early stage disease in the *Ldlr* ^*-/-*^ model, and the other a later stage *ApoE* ^*-/-*^ murine model. In experiments with *Ldlr* ^*-/-*^ mice we investigated whether there was a time-dependent increase in the relative number of *Tcf21* expressing cells that were undergoing cell division. In these studies, *Tcf21* ^*iCre/+*^, *ROSA* ^*tdT/+*^, *Ldlr* ^*-/-*^ mice were treated with tamoxifen at 1 week before isolation, and EdU was injected 24 hours prior to tissue harvest at relatively early disease time points for this model, at 0, 9, 12, and 15 weeks of feeding with HFD. EdU positive cells were found in the adventitia, media and intima of the aortic wall and quantified in sections of the aorta through the region of the sinus. The percentage of proliferating *Tcf21* expressing cells was identified by both tdT and EdU fluorescence (Fig 7A). Comparison of the number of *Tcf21*+EdU+ cells versus all *Tcf21*+ cells at different time points showed a significantly greater percentage of proliferating cells at 12 weeks of diet compared to time zero (1.94±0.70 vs. 6.42±1.10, *P*<0.05) (Fig 7B). There was also an increase in the mean percentage of EdU+ *Tcf2*1+ cells to EdU+ cells between the 9 and 12 week time points, although this difference did not reach statistical significance (*P* = 0.053). Studies also investigated whether there was a difference in the relative rate of cell division in lesions of *Tcf21* ^*lacZ/+*^, *ApoE* ^*-/-*^ mice compared to *ApoE* ^-/-^ animals, after 20 weeks of HFD. All mice were injected with EdU 24 hours prior to tissue harvest and the number of EdU fluorescent labeled cells was compared to the total number of cells as identified by DAPI fluorescence for each genotype. This analysis was restricted to the plaque area where the majority of EdU+ cells were located in these more mature lesions (Fig 7C and 7D). The *Tcf21* ^*lacZ/+*^, *ApoE* ^*-/-*^ mice showed a significantly lower number of EdU+ cells compared to *ApoE* ^-/-^ animals (2.25±0.18 vs. 3.27±0.25, P<0.05) (Fig 7E). Taken together, these data suggest that *Tcf21* expressing cells show a time-dependent increase in proliferation with disease progression, possibly greater than other cells not expressing *Tcf21*, and that this increase may depend on *Tcf21* expression.

### TCF21 is differentially expressed in the fibrous plaque of ruptured human atherosclerotic lesions

**Fig 8:**
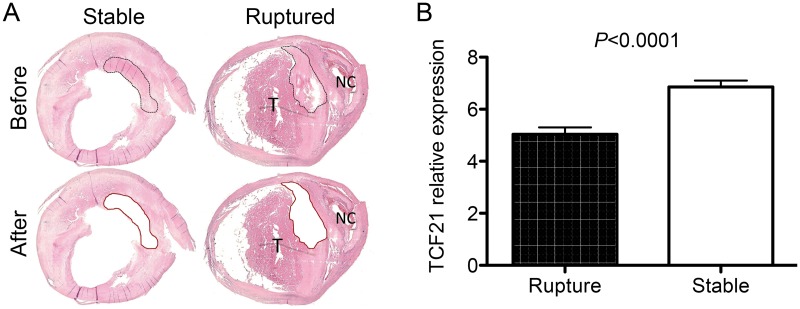
Differential *TCF21* gene expression in fibrous cap of stable vs. ruptured atherosclerotic plaque. A) Microdissection laser capture was employed to harvest tissue from endarterectomy samples from vessels with stable plaque or evidence of plaque rupture. An example of a stable plaque and a ruptured plaque with necrotic core (NC) and related luminal thrombus (T) are shown. These representative sections show the types of plaque regions that were harvested for RNA isolation. B) qPCR quantitation of *TCF21* mRNA levels in harvested tissue. TCF21 expression was significantly lower in fibrous cap tissue from the ruptured plaque.

To begin to investigate the possible association of *TCF21* expression and function in the fibrous cap of human atherosclerotic lesions, we have investigated the *TCF21* expression pattern in fibrous plaque of carotid atherosclerotic lesions collected from patients at time of endarterectomy surgery. Micro-dissection laser capture was used to obtain tissue from the fibrous cap of 10 vessels that showed stable plaque morphology and 10 vessels that had undergone plaque rupture (Fig 8). RNA was isolated from these tissues and employed to assess *TCF21* mRNA levels by qPCR. This analysis showed significantly decreased levels of *TCF21* expression in the fibrous cap tissue harvested from the ruptured plaque (5.0±0.3 vs 6.9±0.3, ruptured vs stable plaque, *P*<0.0001).

## Discussion

SMC are critical contributors to atherosclerosis pathophysiology, and relevant to the findings reported here is the dramatic alteration in gene expression and basic cellular functions that occur with phenotypic modulation of this cell type in the disease setting [5,32,33]. In the classic response to injury hypothesis, fully differentiated medial SMC express contractile marker genes and have limited proliferative capacity, while under disease conditions they downregulate contractile gene expression, proliferate, and migrate into the neointimal space. Data presented here suggest that upregulation of *TCF21* may be directly responsible for at least some aspects of the phenotypic modulation that is believed to characterize the medial SMC response to vascular injury. We have shown that TCF21 binds the *ACTA2* locus, transrepresses *ACTA2* reporter genes in cultured SMC, and that knockdown of *TCF21* is associated with increased *ACTA2* expression. Although we have not studied other SMC markers in such detail, both *TAGLN* and *MYH11* are upregulated in si*TCF21* treated cells. While additional studies are required to characterize a possible dedifferentiation function for *TCF21*, also consistent with such a hypothesis are *in vitro* studies in cultured HCASMC presented here which show that *TCF21* promotes proliferation and migration. A differentiation inhibiting function has previously been proposed for *TCF21* in skeletal muscle cells [34].

*Tcf21* is expressed in epicardial progenitor cells that give rise to coronary SMC, and its expression in the adult might be characteristic of a precursor cell that is resident in the vascular wall and is capable of giving rise to SMC in the setting of vascular injury. A number of previous studies have identified such precursor cells in distinct compartments of the vessel wall, all with some capacity to give rise to differentiated SMC. It is generally accepted that SMC accumulation in the fibrous cap is monoclonal or oligoclonal, and this is consistent with the existence of a resident arterial subpopulation of SMC precursor cells that contribute to healing in the response to disease stresses [35]. In bovine and canine vessels, subsets of medial SMC have prominent proliferation with reduced SMC marker expression, and this population has been estimated to be 6–15% of cells in the murine aorta [36–38]. These observations are consistent with data presented here showing that *Tcf21* expressing cells with low SMC marker expression are distributed in the proximal aorta in mouse. Cells with SMC precursor function have also been identified in the adventitial layer [35]. In mouse, Sca1^+^ cells with SMC differentiation potential have been identified in the aortic adventitia [39], and in humans, a population of cells with SMC potential has been identified in the space between the media and adventitia of large and medium sized vessels [40]. While there are reported differences in the features and markers of these and other progenitor-like cells in the vascular wall, here we demonstrate an overlap of expression pattern and other features in *Tcf21* expressing cells. It seems likely that *TCF21* may be expressed in one or more of these cell populations and may contribute to an undifferentiated SMC precursor phenotype.

The studies described herein provide important new information regarding the biology of the fibrous cap. While the risk of plaque rupture appears to be inversely correlated with the number of SMC in the fibrous cap, there is limited understanding of their *in vivo* origin, and the molecular pathways that regulate their expansion and terminal phenotype determination [5,41]. With these studies we have shown *Tcf21* to be a marker for precursor cells that give rise to at least a portion of fibrous cap cells, and genomic studies in conjunction with *in situ* protein marker studies identified a number of signaling molecules that relate to the phenotype of this important cell type. While Pdgfra and Pdgfrb have been previously identified on fibrous cap cells [42,43], we show here that they are not expressed by cells that are migrating toward the cap and expressing *Tcf21* but initiate expression in conjunction with upregulation of SMC marker genes when they are juxtaposed to the endothelium. Tgfbr2 has not previously been identified on fibrous cap cells, and its biology in this context not studied, but it has recently been shown to support the differentiated SMC phenotype and homeostasis of this cellular lineage [27,28]. *TGFBR2* mRNA levels were found to be regulated by *TCF21* in the siRNA knockdown RNA-Seq studies. Another protein expressed by *Tcf21* lineage-traced cells in the fibrous cap, periostin, is known to promote SMC differentiation and migration [44–46], and interestingly has recently been associated with the atherosclerotic phenotype in young humans [47]. Additional follow-up of genomic studies are expected to provide insights into other genes and pathways that are present in *TCF21* marked fibrous cap precursor cells.

Lineage tracing provides a unique and informative approach to address questions related to cellular origin, movement and differentiation of lesion cells in the vascular wall, however a number of questions remain unanswered. Given the capacity of *Tcf21* to promote autonomous cellular proliferation and migration, and the appearance of lineage traced cells in lesions of animals where recombination preceded HFD feeding, it seems likely that lineage traced cells can migrate from the adventitia or media to contribute to lesion formation. Although there is some evidence that bone marrow—derived SMC-like cells may contribute to neointima formation, such cells appear to represent a very small fraction of the total neointimal cells [48,49]. Given the variability in disease progression in different *ApoE* ^*-/-*^ and *Ldlr* ^*-/-*^ animals, and the possibility that *Tcf21* is upregulated in adventitial or medial cells throughout disease progression, lineage tracing does not allow assessment of how much of the fibrous cap derives from *Tcf21* expressing cells. Results from the *Tcf21* ^*lacZ/+*^, *ApoE* ^*-/-*^ studies suggest that *Tcf21* expressing cells constitute a significant portion of the lesion SMC but this cannot be readily evaluated with current methods. Also, evidence that some fibrous cap SMC derive from lineage traced cells does not eliminate the possibility that a portion of the fibrous cap cells continue to express *Tcf21* and maintain a fibroblast or myofibroblast phenotype, and that the fibrous cap is actually a combination of such cells along with those that have downregulated *Tcf21* and adopted a true SMC phenotype. This possibility is consistent with sustained *Tcf21* expression in some of the fibrous cap cells at the 20-week disease timepoint (Fig 3).

While the work reported here has provided important initial details of how this gene is expressed during atherosclerotic vascular disease and the types of processes that it might regulate to affect disease pathophysiology, the causal mechanisms of disease risk remain unclear. The direction of effect has not been fully established by current available human genetic and genomic data, with both directions being suggested by different approaches and possible underlying gene regulatory mechanisms characterized for each [8,50,51]. Based on the animal model findings reported here, greater *TCF21* expression could inhibit disease risk by promoting the expansion of SMC lineage and contributing to plaque stabilization by supporting fibrous cap development. Alternatively, greater *TCF21* expression in SMC precursor cells at the fibrous cap might inhibit their differentiation to the SMC lineage, thus destabilizing the plaque. Similar alternative hypotheses could be generated for the risk allele producing decreased *TCF21* expression. Nonetheless, further *in vivo* gain- and loss-of-function studies are required to determine how changes in *Tcf21* gene expression affects the size and architecture of the fibrous cap and how such changes correlate to specific human disease traits linked to plaque vulnerability and rupture. These questions could be addressed using the various plaque rupture models established in mice [52,53]. Finally, it will be important to investigate the phenotype of the *TCF21* expressing cells at baseline, and how the phenotype changes as these cells migrate into the lesion. Such studies could be performed with captured cells using modern genomic methods, and would provide extremely valuable insights into the cellular phenotype as these cells respond to the vascular signals of the disease process.

## Methods

### Ethics statement

All procedures described in this study were approved by the Institutional Animal Care and Use Committees of Stanford University and University of Hawaii and conformed to NIH guidelines for care and use of laboratory animals.

### Cell culture

Human primary Coronary Artery Smooth Muscle Cells (HCASMC, #CC-2583) were obtained from Lonza (Allendale, NJ, USA) at passage 4 and cultured in Smooth Muscle Growth Medium-2 including hEGF, insulin, hFGF-B and FBS, but without antibiotics (Lonza, #CC-3182). Human embryonic kidney cells (HEK, ATCC #CRL-11268) and rat aortic smooth muscle cells (A7R5, ATCC #CRL-1444) were cultured in DMEM High Glucose (Life Technologies #311995–065) with 10% FBS (Life Technologies #26140–79). Human cardiac fibroblast (HCF), bone-marrow derived mesenchymal stem cells (bmMSC), embryonic stem cell derived mesenchymal stem cells (eMSC), induced pluripotent stem cell-derived mesenchymal stem cells (iMSC), human aortic adventitial fibroblasts (HAoAF), human aortic endothelial cells (HAoEC), human coronary artery endothelial cells (HCAEC), and human aortic smooth muscle cells (HAoSMC) were obtained from commercial suppliers, maintained in the recommended media, and evaluated with at least 3 technical replicates.

For RNA-Seq studies and individual gene quantitative PCR, HCASMC were transfected with 300 nM *TCF21* Trilencer-27 Human siRNA (OriGene #SR304753C) or Trilencer-27 Universal Scrambled Negative Control siRNA (OriGene #SR30004) at 80% confluence using the Amaxa Basic Nucleofector Kit for Primary Mammalian Smooth Muscle Cells (Lonza #VPI-1004) at a density of 1 x 10^6^ cells per 100 μL sample using Nucleofector Program U-025. Cells were changed to medium with supplements at 18 hours post-transfection and cultured for an additional 48 hours.

### Lentivirus construction and in vitro cell culture assays

For *TCF21* overexpression, a cDNA clone (OriGene #SC125048) representing the protein coding sequence of human *TCF21* was cloned into pWPI vector (Addgene #12254) which also contains the GFP reporter gene. For shRNA knockdown the following sequences were cloned into pLVTHM (Addgene #12247):

#### sh*TCF21* construct 1

1:GaattcgaacgctgacgtcatcaacccgctccaaggaatcgcgggcccagtgtcactaggcgggaacacccagcgcgcgtgcgccctggcaggaagatggctgtgagggacaggggagtggcgccctgcaatatttgcatgtcgctatgtgttctgggaaatcaccataaacgtgaaatgtctttggatttgggaatcttataagttctgtatgagaccacagatctCCCTGGATTCGAACAAGGAATTTGTGACTTTTCAAGAGAAAGTCACAAATTCCTTGTTCGAATCCATTTTTGGAAaagcttATCGAT

#### sh*TCF21* construct 2

GaattcgaacgctgacgtcatcaacccgctccaaggaatcgcgggcccagtgtcactaggcgggaacacccagcgcgcgtgcgccctggcaggaagatggctgtgagggacaggggagtggcgccctgcaatatttgcatgtcgctatgtgttctgggaaatcaccataaacgtgaaatgtctttggatttgggaatcttataagttctgtatgagaccacagatctCCCTGGAGATGTTGGAATGTGACGGGTTGATTCAAGAGATCAACCCGTCACATTCCAACATCTCCATTTTTGGAAaagcttATCGAT

Lentiviruses were produced by polyethylenimine (Polysciences cat #23966–2)-mediated transfection of 293T cells (American Type Culture Collection #CRL-11268). Per 225 cm^2^ flask, 3.6 x 10^7^ cells were plated 24 hours before transfection in DMEM (Invitrogen cat #11995), 10% fetal bovine serum (Hyclone cat #SH30070.03). For transfection, 30 mg of lentivirus vector, 30 mg of a HIV-1 gag-pol-rev-tat helper plasmid (pCMV DR8.9, Addgene #8455), and 15 mg of a plasmid encoding the VSV-G envelope protein (pMD2.G, Addgene #12259) were used per T-225 flask of cells transfected. The plasmids (75 mg total) were added to OptiMEM (Invitrogen cat # 31985) such that the total volume was 700 ml and then 300 ml of 1 mg/ml polyethylenimine (25 kDa linear, pH 4.5 in 1 x PBS) was added. The PEI/DNA mixture was mixed, incubated 10 min at 20°C, and then added to the cells. The cells were incubated at 37°C in a 5% CO_2_ atmosphere. Then 24 and 48 hours after transfection the media was harvested and replaced with fresh media. After harvesting the media, dead cells were removed by centrifugation at 3,000 x g for 15 min. The virus was filtered through a 0.45 μm Steri-flip filter (Millipore cat #SE1M003M00) and then aliquoted and stored at -80°C until use.

HCASMC were transduced with lentiviral particles at a MOI of 22.5 at passage 7 to 9. The transduction efficiency was assessed by quantifying the percentage of GFP-positive cells by flow cytometry on a BD FACSCalibur. Transduction efficiencies were between 50 and 95 percent.

#### Cell migration (gap closure) assay

The gap closure assay (Cell Biolabs #CBA-126) was conducted according to the manufacturer’s protocol. Briefly, 10,000 lentivirus transduced cells were seeded per well and let attach overnight. After gel removal 3 wells per condition were directly stained with crystal violet and imaged using a Zeiss Axioplan 2 instrument using the NIS-Elements F v4.0 software while the remaining 9 wells per condition were incubated for 12h before crystal violet staining. The covered area per well was quantified using ImageJ v1.47.

#### In vitro proliferation assay (cell counting)

Lentivirally transduced HCASMC were plated into 6 well plates at low density (10,000 cells per well, 3 wells per condition) and harvested at given time points before reaching confluence. Viable cell numbers were then counted manually and percentage of GFP positive cells measured by flow cytometry.

#### In vitro proliferation assay (EdU labeling)

Lentivirus transduced cells were plated to a 12 well plate at sub-confluence density and allowed to recover overnight. The cells were then serum starved for 24 hours. Following starvation, the cells were exposed to serum for 24 hrs, with treatment during the last 3 hrs of this period with EdU from the Click-iT EdU Alexa Fluor 488 Imaging Kit (Life technologies, Carlsbad, CA; Cat# C10377) at a concentration of 20uM. The cells were incubated with EdU for 3 hours, then fixed and permeabilized using 4% PFA and 0.5% Triton-X in PBS, respectively. This was followed by incubation with Click-iT reaction cocktail, including CuSO4 and Alexa Fluor Azide, and then with nuclear staining with DAPI solution. Using a Leica inverted microscope, the number of total nuclei, and the number of co-stained nuclei were counted using the ImageJ (NIH) software on ten consecutive 10x fields for each condition.

#### In vitro apoptosis assay

Cells were transfected with siControl or si*TCF21* reagents exactly as described above for the RNA-Seq studies, at a density of 1E6 cells per 100 μL sample using Nucleofector Program U-025. Cells were changed to medium without supplements at 18 hours post-transfection and cultured for an additional 48 hours. Caspase activity in these cells was detected using the Caspase-Glo 3/7 Assay (Promega #G8093).

### RNA isolation and quantitative real-time RT-PCR

For qPCR RNA was isolated using QIAzol (Qiagen #79306) and the Trizol RNA isolation protocol. DNA was removed with the DNA-free kit from Ambion (Life Technologies #AM1906) followed by first strand cDNA synthesis and subsequent RNA digestion according to the SuperScript III protocol (Life Technologies #18080–051). Relative gene expression was measured using the ViiA 7 Real-Time PCR System from Applied Biosystems (Life Technologies). cDNA was amplified as follows: 95°C 15 min, 40x (95°C 1s, 60° 20s). Locus enrichment in chromatin immunoprecipitation samples was measured using the ABgene SYBR mastermix (AB-1166) and the primers ACTA2chipfor 5’-AGGGAGATGCAAACCAGATATCC-3’, ACTA2chiprev 5’-GCAGGTGACCTGCTGAATTTTTC-3’, PROCRchipfor 5’-GACTCCTGCTTACCTCCTCATA-3’ and PROCRchiprev 5’-GGGTGAAGAAGGTACAAAAGAA-3’. Genomic DNA cycling conditions were 95°C 15min, 40x (95°C 15s, 60°C 1 min). Absolute quantification of expression levels was performed using a standard dilution series and normalization to 18S rRNA. Relative expression levels were then calculated relative to *TCF21* expression levels in HEK cells (S1A Fig) or in SiCTRL treated HCASMC (Fig 2E) by division.

### RNA sequencing

Total RNA from either si*TCF21* or siCTRL treated samples was depleted for ribosomal RNA with the Ribo-Zero magnetic kit from Epicentre (Illumina #MRZH116), libraries generated with the Epicentre ScriptSeq v2 RNA-Seq library preparation kit (Illumina #SSV21106) and thereafter sequenced as 100bp paired-end reads on an Illumina HiSeq 2000 instrument. The resulting data has been deposited at GEO under accession number GSE44461.

Reads resulting from RNA-Sequencing of siCTRL and si*TCF21* treated HCASMC were mapped using software tools TopHat+Bowtie2. Differential expression level between samples was analyzed using the software tools DESeq and edgeR at an FDR ≤ 0.05, with intersection of the 466 and 430 respective identified genes providing a group of 380 common genes. This genelist was used to interrogate the Ingenuity Knowledge Base, identifying over-representation of Cardiovascular Disease annotation terms, including the top functional category “Vascular disease.” Gene members of this group were expanded by adding 18 genes with the “build” function in IPA to create the TCF21 Vascular Disease Network. Visualization of the network was performed using Cytoscape open source software. Node color was mapped to log fold change (green-yellow-red palette), node size to absolute expression value in wild type cells, and font size to enrichment *Q*-value. GO categories were assigned to genes using the Bingo application for Cytoscape and coloring the GO categories was performed using GOlorize application for Cytoscape.

### Mouse models

A *Tcf21* functional knockout and reporter line, *Tcf21* ^*lacZ/+*^, was constructed by substituting the *lacZ* gene into the first exon of the murine gene employing homologous recombination in embryonic stem cells, as described previously [12]. Fidelity of expression of the *lacZ* reporter has been described [12], and methods for using Xgal staining to track *Tcf21* expression were identical to those published previously from this laboratory [54]. For studies of reporter gene expression in the *ApoE* ^*-/-*^ model, animals were weaned onto Western high fat diet (HFD, 21% anhydrous milk fat, 19% casein and 0.15% cholesterol, Dyets no. 101511). The animals were euthanized and perfused with PBS followed by 0.4% PFA, the aortic roots excised and subjected to Xgal staining followed by post-fixation for 30 minutes in 4% PFA. After incubation in an ascending sucrose solution series and embedding in OCT, tissue was processed into 7 μm thick frozen sections for immunohistochemistry.

Lineage tracing studies employed a recently described inducible *Tcf21*-Cre line (*Tcf21* ^*iCre/+*^) [31], and tomato (B6.Cg-*Gt(ROSA)26Sor* ^*tm14(CAGtdTomato)Hze*^/J) Jax #007914 and *lacZ* (B6.129S4-*Gt(ROSA)26Sor* ^*tm1Sor*^/J) Jax # 003474 reporter lines. The *Tcf21* ^*iCre/+*^ line was obtained on a mixed C57Bl/6 x 129SV mixed background and was bred back onto the C57Bl/6 background for 6 generations before being mated with the *ApoE* ^*-/-*^ line. The *Tcf21* ^*iCre/+*^ allele was bred directly onto the *Ldlr* ^*-/-*^ line in C57Bl/6 background. All *Ldlr* ^*-/-*^ mice were treated with a modified Western high fat diet (HFD, 15.8% milk fat and 1.25% cholesterol (diet 94059; Harlan Teklad) for the time periods indicated. *Tcf21* ^*iCre/+*^, *ROSA* ^*tdT/+*^, *ApoE* ^*-/-*^; *Tcf21* ^*iCre/+*^, *ROSA* ^*tdT/+*^, *Ldlr* ^*-/-*^; and *Tcf21* ^*iCre/+*^, *ROSA* ^*lacZ/+*^, *ApoE* ^*-/-*^ and *Ldlr* ^*-/-*^ and *ApoE* ^*-/-*^ control animals were treated as described with HFD, tissues harvested and sectioned as described above, and analyzed for expression of the reporter genes as well as for other cellular markers as described below. For *ApoE* ^*-/-*^ animals, Cre recombination was activated with a series of ten 1 mg tamoxifen (Sigma Aldrich) intraperitoneal injections for a total of 10 mg of tamoxifen. Animals were induced at 0, 6, 10 weeks and sacrificed after 20, 8, and 12 weeks respectively of the HFD as described above. For *Ldlr* ^*-/-*^ mice, Cre induction was accomplished by feeding of tamoxifen chow at 3–5 weeks of age (Harlan Laboratories, Inc).

### Immunostaining

For immunocytochemistry, HCASMC were seeded into culture slides (BD Biosciences #35641) in supplemented medium for 24 hours. For immunohistochemistry of human tissues, paraformaldehyde-fixed human coronary arteries were frozen, embedded and sectioned at a thickness of 7 μm and tissue slides were postfixed with 4%PFA in PBS for 10 minutes. Staining for TCF21 expression was conducted on frozen tissue sections with a polyclonal anti-TCF21 antibody (Sigma-Aldrich #HPA013189) at a dilution of 1:100, with overnight primary incubation.

Mouse tissues were collected and processed as described above. After postfixation, nonspecific binding sites were blocked for 30 minutes with Rodent Block M (Biocare Medical #RBM961). Primary antibody incubation was performed overnight at 4°C using the following antibodies and dilutions in Da Vinci Green antibody diluent (Biocare Medical #PD900), anti-Acta2 1:300 (Abcam #ab5694), anti-Tagln 1:300 (Abcam ab14106), anti-Pdgfrb 1:500 (Abcam #ab32570), anti-Tgfbr2 1:100 (Abcam #ab61213), anti-periostin 1:200 (Santa Cruz #sc49480), anti-Pdgfra (R&D Systems #AF1062), anti-vWF 1:250 dilution (Abcam ab11713), anti-TUBB3 1:1000 dilution (Abcam ab78078), anti-PLIN1 1:500 dilution (Abcam ab61682), anti-THY1 1:200 dilution (Dianova DIA-100-M), anti-NES 1:2000 dilution (Abcam ab134017), anti-LAMP2 1:150 dilution (Abcam ab37024), anti-PTPRC 1:300 dilution (Abcam ab), and anti-ITGAX 1:100 dilution (Abcam ab33483). The slides were incubated for 1h at room temperature with Rabbit-on-Rodent AP polymer (Biocare Medical #RMR625) and 10–15 minutes with the substrate Vulcan Fast Red (Biocare Medical #FR805). Between steps slides were washed 2 x 5 minutes with TBS (Sigma-Aldrich #T6664). The slides were then dehydrated in an ascending alcohol dilution series and mounted in Cytoseal XYL (Thermo Scientific #8312–4). Images were taken with a Zeiss Axioplan 2 using the NIS-Elements F v4.0 software.

EdU staining was employed to quantify in vivo rates of vascular wall cell proliferation in *Tcf21* ^*iCre/+*^, *ROSA* ^*tdT/+*^, *Ldlr* ^*-/-*^ mice. These mice were weaned onto HFD at 4 weeks of age, administered tamoxifen by a single gavage of 10 μg/40g body weight 1 week before tissue harvest, and sacrificed and evaluated at T0, 9wks, 12 wks or 15 wks of diet. 5-Ethynyl-2-deoxyuridine (Santa Cruz, 50μg/g body weight) was injected intraperitoneal 24 hours before sacrifice. Aortic root tissues were harvested, processed and sectioned, and *Tcf21*+EdU+ positive cells were quantified and expressed as the % of *Tcf21* lineage cells. This percentage was evaluated for significant differences among the various time points evaluated. These data are based on three 40x images at each time point in each animal.

Similar experiments were performed to compare the relative rates of cell division in *Tcf21* ^*lacZ/+*^, *ApoE* ^*-/-*^ compared to *ApoE* ^*-/-*^ mice. Animals in each group were injected with 1mg of EdU (Life Technologies, cat#A10044) 24 hours before sacrifice and EdU incorporation was imaged according to the manufacturer's instructions with the Click-iT EdU Alexa Fluor 488 Imaging Kit (Life Technologies) on OCT (Tissue-Tek) frozen sections. Eight sections were assessed from each group with ImageJ software (NIH) to determine the ratio of EdU positive cells to the total number of DAPI positive cells within plaque areas.

### Micro-dissection laser capture and TCF21 expression analysis in human atherosclerotic lesions

Human atherosclerotic carotid artery lesions were obtained from patients undergoing endarterectomy surgery for stable (asymptomatic; *n* = 10) or vulnerable (symptomatic; *n* = 10) carotid stenosis, as part of the Biobank of Karolinska Endarterectomies (BiKE). Samples were collected with informed consent procedure and the study approved by the Local Ethical Committee. Patient demographics, symptom definition and sampling routines were described previously [55]. Micro-dissection laser capture of fibrous cap structures from both subgroups of patient samples was performed using a PALM Microlaser system (Zeiss, Germany) according to manufacturer’s instruction. Atherosclerotic lesions, stable and ruptured (vulnerable), were paraffin-embedded, sectioned, and stained with hematoxylin & eosin on RNAse-free glass slides. Sections were pre-treated with UV light at 254nm for 30 minutes to overcome the hydrophobic nature of the membranes and to enhance adhesion of the paraffin-embedded sections. Ten consecutive slides per individual patient were micro-dissected and then pooled for gene expression analysis.

In preparation for laser pressure catapulting, sections were first de-paraffinized with Xylene (2 x 2 minutes) and decreasing C_2_H_6_O (Ethanol) concentrations (100%, 96%, 70% each for 1 minute). Sections were then rinsed in RNAse-free water, and stained for 10 minutes with Mayer’s hematoxylin (Sigma, USA), again rinsed for 3 minutes in RNAse-free water, and then consecutively stained for 3 minutes with Eosin (Sigma, USA). Finally, samples were dehydrated with increasing concentrations of C_2_H_6_O, before briefly being air-dried at room temperature. After micro-dissection and catapulting, the fibrous cap sample was collected into AdhesiveCaps (Zeiss, Germany) and 350μl of RLT buffer (Qiagen, Germany) was added, and mixed by inversion after closure. The lysate was spun down for 5 minutes at 13 000 rpm and stored at -80°C.

RNA extraction from catapulted, micro-dissected samples was performed using the RNeasy Micro Kit (Qiagen, Germany) following the manufacturer’s protocol. RNA was quantified by Nanodrop (Agilent Technologies), and RNA quality was verified using an Agilent 2100 Bioanalyzer (Agilent Technologies). Samples required 260/280 ratios >1.8, and sample RNA integrity numbers >9 for inclusion. The TaqMan High Capacity cDNA Transcription Kit was used for cDNA synthesis, and primer assays for *TCF21* and 18S (for housekeeping/endogenous control) were utilized to detect changes in mRNA expression levels. The mean *TCF21* mRNA level (run in triplicate) was normalized to the mean 18S mRNA level (run in triplicate) to calculate the change in expression for each condition.

### Statistical analysis

Results are expressed as means ± standard deviation (SD) with number (n) of replicates. Statistical comparisons of two groups were performed by two-tailed t-test using Prism if not stated otherwise. Welch’s Correction for unequal variance was applied.

## Supporting Information

S1 Fig *TCF21* is expressed by coronary vascular cells. A) Relative *TCF21* expression levels in human cultured cells as measured by quantitative realtime RT-PCR: HEK, human embryonic kidney cell line; HCF, human cardiac fibroblast; bmMSC, bone-marrow derived mesenchymal stem cells; eMSC, embryonic stem cell-derived mesenchymal stem cells; iMSC, induced pluripotent stem cell-derived mesenchymal stem cells; HAoAF, human aortic adventitial fibroblasts; HAoEC, human aortic endothelial cells; HCAEC, human coronary artery endothelial cells; HAoSMC, human aortic smooth muscle cells; HCASMC, human coronary artery smooth muscle cells. Numbers for HCASMC data represent different donor samples. B) Immunostaining for TCF21 (green) and ACTA2 protein (red) in cultured HCASMC. A number of prominent cells expressing high levels of ACTA2 appeared to have low-level expression of TCF21. *Tcf21* anti-sense transcript in situ hybridization was conducted with C) mouse tissue sections and *TCF21* hybridizations conducted with D) sections of human coronary artery tissues, revealing high level of specific (blue) labeling of adventitial cells. There was no evidence of staining of the medial or endothelial cell layer. E) Control hybridizations were performed with species relevant sense transcripts. F) *Tcf21* ^*lacZ/+*^ reporter mice were employed with Xgal *in situ* staining to investigate *Tcf21* expression in the adult cardiovascular system. The low power view at left is evaluated with Xgal cytochemical staining (blue) and Acta2 immunostaining (red). The boxed area is localized on the coronary artery and is visualized in panels to the right at high power. β-galactosidase enzymatic activity was localized primarily to the adventitia, with some *Tcf21* expressing cells being located adjacent to the external elastic lamina in juxtaposition to the medial SMC and other cells being localized to the loose adventitial tissue more distantly separated from the vascular wall. Combined immunostaining for Acta2 (red) expression and β-galactosidase activity (pseudocolored green) did not show colocalization (yellow color) and suggested that *Tcf21* expressing cells did not express this SMC marker. G) The low power view at the left shows tissue at the aortic root, evaluated with Xgal cytochemical staining (blue) and Acta2 immunostaining (red). The boxed area is localized on the aortic wall and is visualized in panels to the right at high power. *Tcf21* expression visualized as β-galactosidase activity was observed in proximal aortic medial cells in a patchy distribution, with no apparent overlap in expression for *Tcf21* and Acta2 as would be shown with yellow color.
(TIF)

S2 Fig si*TCF21* knockdown for RNA-Seq studies. A) si*TCF21* transfected into HCASMC provided a significant decrease in mRNA levels for *TCF21*, (0.99±0.02 control vs. 0.25±0.02 si*TCF21*, *P*<0.0001). B) Western blot of protein extracts from HCASMC that were mock transfected compared to those that were transfected with control siRNA or si*TCF21*. Quantitation of blots showed a decrease in protein level to 26% of baseline in the cells treated with si*TCF21* compared to siCTRL.
(TIF)

S3 Fig Gene ontology of the TCF21 Vascular Disease Network derived from RNA-Seq studies of HCASMC exposed to *TCF21* knockdown. Differentially regulated genes were employed to construct an interaction network highlighting the gene ontology (GO) annotation information of the network genes. Visualization of the network was performed in Cytoscape. Molecular function gene ontology terms were assigned to the network nodes using the Bingo Cytoscape application and colored with GOlorize Cytoscape. Log values of the relative expression level fold changes are represented in a green-red color palette as a circle surrounding the nodes (red up, green down), unless the gene was not assigned with GO terms in which case fold change is the color of the node. Edges were distinguished as described for Fig 1.
(TIF)

S4 Fig Lentiviral overexpression and shRNA knockdown for in vitro studies in SMC. Control lentiviral vectors (pWPI) and lentiviral overexpression vectors (pWPI-*TCF21*), and control (pLVTHM) and lentiviral shRNA mediated knockdown vectors (pLVTHM-sh*TCF21*) were used to transduce primary cultured HCASMC. A) pWPI-*TCF21* increased *TCF21* mRNA levels (1.0±0.04 pWPI vs. 32.5±0.02 pWPI-*TCF21*, P<0.0001), and pLVTHM-sh*TCF21* decreased expression (1.0±0.06 pLVTHM vs. 0.34±0.04 pLVTHM-sh*TCF21* 2, P<0.001). B) Western blots of protein extracts from HCASMC that were transduced with over-expression and knockdown lentiviruses showed a 4.5-fold increase, and reduction of TCF21 protein levels to 8% (sh*TCF21* 1, sh*TCF21* 2) of baseline respectively.
(TIF)

S5 Fig *TCF21* regulates cell division in vitro in HCASMC. A) Flow cytometry of cultured HCASMC transduced with *TCF21* overexpressing lentivirus (pWPI-*TCF21*) or empty lentivirus (pWPI empty), both of which express GFP, was employed to evaluate how *TCF21* affects cell division. HCASMC showed an increase in *TCF21* overexpressing cells from 48 to 82 percent of the culture within 25 days. B) Similar knockdown experiments were conducted with shRNA expressing lentiviruses (sh*TCF21* 1, sh*TCF21* 2) as well as the parent pLVTHM which served as control. All vectors expressed GFP. There was a significant decrease in GFP positive cells at day 28, si*TCF21* 1 vs. siCTRL *P*<0.0001; si*TCF21* 2 vs. siCTRL *P*<0.0001. Taken together, these data are consistent with a pro-proliferative role for *TCF21*.
(TIF)

S6 Fig TCF21 and ACTA2 immunohistochemistry of human vascular tissues with varying degrees of disease severity. A-C) In vessels with minimal disease there was adventitial TCF21 staining (red) with some patchy staining of the minimal neointima (red arrows), but no staining in the media which was positive for ACTA2 (brown). D, E) In vessels with significant disease there was robust TCF21 staining in the adventitia and neointima and some patchy staining in the fibrous cap, but very little staining in the media. ACTA2 staining was prominent in the fibrous cap and less so in the media. F) Control studies without primary antibodies showed only weak background staining. Lu, lumen; In, neointima; M, media; FC, fibrous cap.
(TIF)

S7 Fig *Tcf21* reporter gene expression in mouse vascular tissues with combined immunohistochemical staining for various cellular lineage markers. Various antibodies were employed for lineage markers with tissue from *Tcf21* ^*lacZ/+*^, *ApoE* ^*-/-*^ animals, Xgal stain is blue and immunohistochemical staining is red for lineage markers.
(TIF)

S8 Fig *Tcf21* expressing cells in *ApoE* ^*-/-*^ lesions give rise to smooth muscle cells in the fibrous cap. *Tcf21* ^*iCre/+*^, *ApoE* ^*-/-*^ mice were administered tamoxifen to activate expression of an inducible MerCreMer construct knocked into the *Tcf21* locus. Cre mediated recombination of a *lacZ* reporter at the constitutively expressed *ROSA26* locus allowed lineage tracing of *Tcf21* expressing cells. Animals received tamoxifen at 6–8 weeks of HFD and tissues were harvested at 12 weeks of diet. A region of atheroma identified by the box in the low power image on the left and shown in high power fields in images on the right show blue β-galactosidase positive *Tcf21* lineage traced cells forming a subcapsular structure. These cells were not labeled by immunostaining for Tagln (red). Similar β-galactosidase positive *Tcf21* lineage traced cells were also seen in association with the fibrous cap and these cells did stain positive for Tagln expression.
(TIF)

S1 Table Cardiovascular disease functional annotation categories derived from si*TCF21* knockdown and RNA-Seq studies of differentially expressed genes. (XLSX)

S2 Table Cardiovascular system development and function annotation categories derived from si*TCF21* knockdown and RNA-Seq studies of differentially expressed genes. (XLSX)

S3 Table Cellular and molecular function terms enriched in "vascular disease" category identified with IPA analysis of si*TCF21* knockdown and RNA-Seq studies of differentially expressed genes. (XLSX)
