# Supplementary figures and images for: Coronary Artery Disease Associated Transcription Factor TCF21 Regulates Smooth Muscle Precursor Cells That Contribute to the Fibrous Cap
Source: PLoS Genet. 2015 May 28;11(5):e1005155. doi: 10.1371/journal.pgen.1005155 (PMC4447275; doi:10.1371/journal.pgen.1005155)

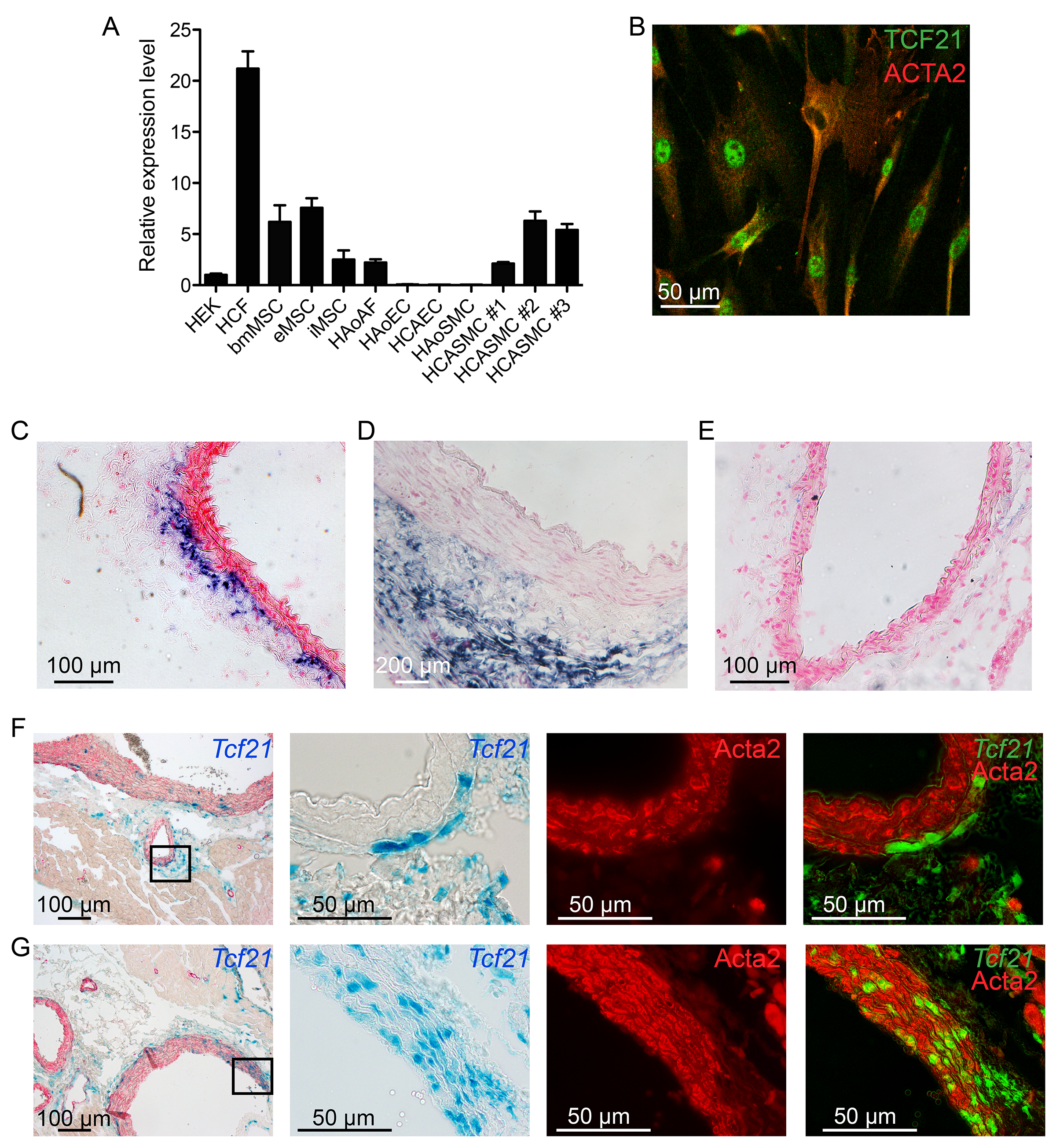

Supplement: S1 Fig — A) Relative TCF21 expression levels in human cultured cells as measured by quantitative realtime RT-PCR: HEK, human embryonic kidney cell line; HCF, human cardiac fibroblast; bmMSC, bone-marrow derived mesenchymal stem cells; eMSC, embryonic stem cell-derived mesenchymal stem cells; iMSC, induced pluripotent stem cell-derived mesenchymal stem cells; HAoAF, human aortic adventitial fibroblasts; HAoEC, human aortic endothelial cells; HCAEC, human coronary artery endothelial cells; HAoSMC, human aortic smooth muscle cells; HCASMC, human coronary artery smooth muscle cells. Numbers for HCASMC data represent different donor samples. B) Immunostaining for TCF21 (green) and ACTA2 protein (red) in cultured HCASMC. A number of prominent cells expressing high levels of ACTA2 appeared to have low-level expression of TCF21. Tcf21 anti-sense transcript in situ hybridization was conducted with C) mouse tissue sections and TCF21 hybridizations conducted with D) sections of human coronary artery tissues, revealing high level of specific (blue) labeling of adventitial cells. There was no evidence of staining of the medial or endothelial cell layer. E) Control hybridizations were performed with species relevant sense transcripts. F) Tcf21 lacZ/+ reporter mice were employed with Xgal in situ staining to investigate Tcf21 expression in the adult cardiovascular system. The low power view at left is evaluated with Xgal cytochemical staining (blue) and Acta2 immunostaining (red). The boxed area is localized on the coronary artery and is visualized in panels to the right at high power. β-galactosidase enzymatic activity was localized primarily to the adventitia, with some Tcf21 expressing cells being located adjacent to the external elastic lamina in juxtaposition to the medial SMC and other cells being localized to the loose adventitial tissue more distantly separated from the vascular wall. Combined immunostaining for Acta2 (red) expression and β-galactosidase activity (pseudocolored gre [file pgen.1005155.s001.tif]

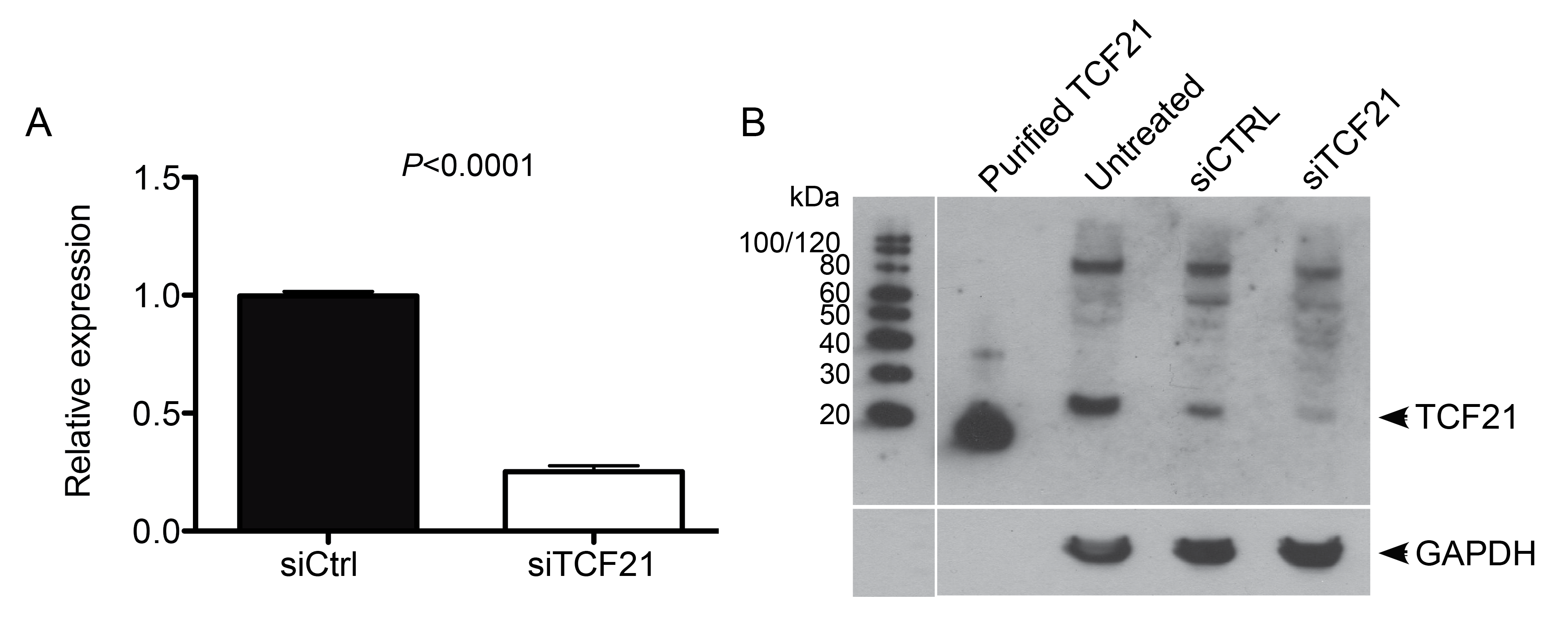

Supplement: S2 Fig — A) siTCF21 transfected into HCASMC provided a significant decrease in mRNA levels for TCF21, (0.99±0.02 control vs. 0.25±0.02 siTCF21, P<0.0001). B) Western blot of protein extracts from HCASMC that were mock transfected compared to those that were transfected with control siRNA or siTCF21. Quantitation of blots showed a decrease in protein level to 26% of baseline in the cells treated with siTCF21 compared to siCTRL. (TIF) [file pgen.1005155.s002.tif]

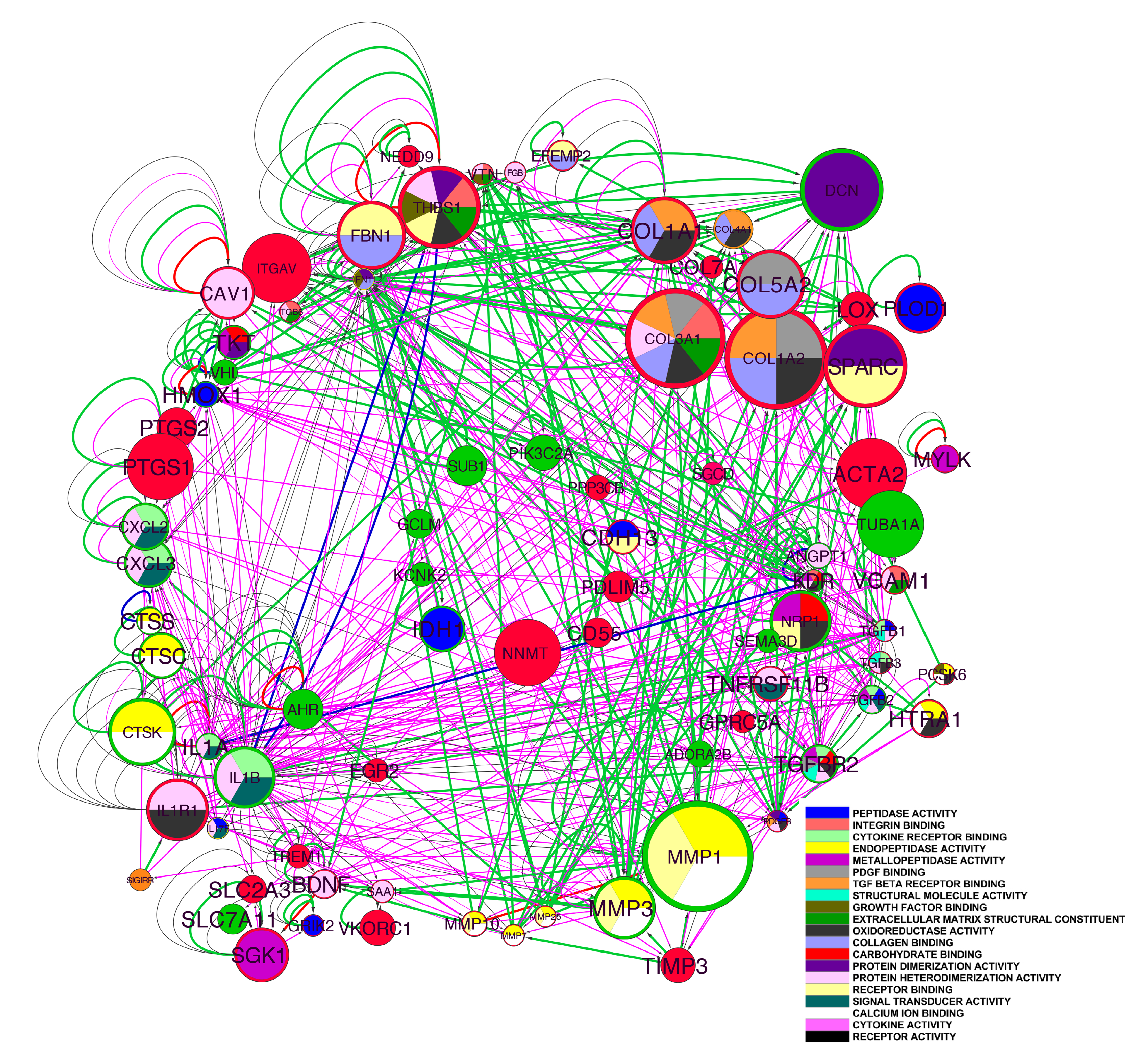

Supplement: S3 Fig — Differentially regulated genes were employed to construct an interaction network highlighting the gene ontology (GO) annotation information of the network genes. Visualization of the network was performed in Cytoscape. Molecular function gene ontology terms were assigned to the network nodes using the Bingo Cytoscape application and colored with GOlorize Cytoscape. Log values of the relative expression level fold changes are represented in a green-red color palette as a circle surrounding the nodes (red up, green down), unless the gene was not assigned with GO terms in which case fold change is the color of the node. Edges were distinguished as described for Fig 1. (TIF) [file pgen.1005155.s003.tif]

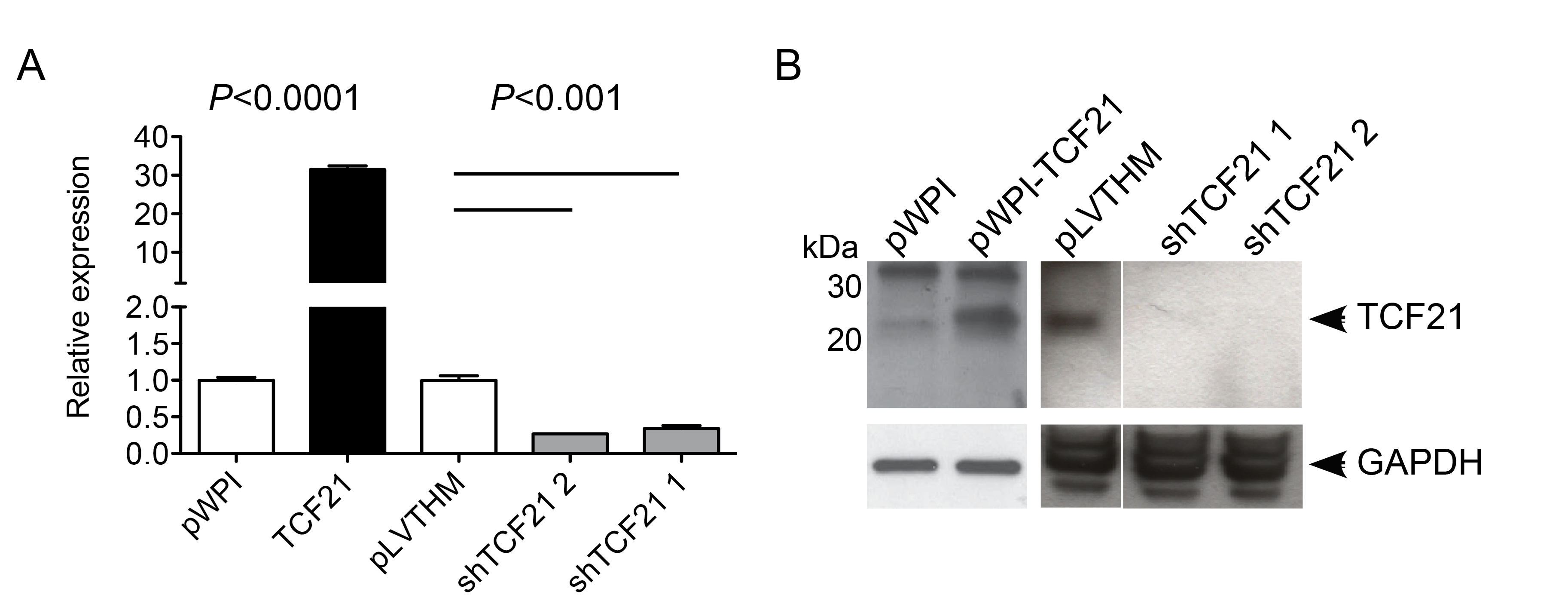

Supplement: S4 Fig — Control lentiviral vectors (pWPI) and lentiviral overexpression vectors (pWPI-TCF21), and control (pLVTHM) and lentiviral shRNA mediated knockdown vectors (pLVTHM-shTCF21) were used to transduce primary cultured HCASMC. A) pWPI-TCF21 increased TCF21 mRNA levels (1.0±0.04 pWPI vs. 32.5±0.02 pWPI-TCF21, P<0.0001), and pLVTHM-shTCF21 decreased expression (1.0±0.06 pLVTHM vs. 0.34±0.04 pLVTHM-shTCF21 2, P<0.001). B) Western blots of protein extracts from HCASMC that were transduced with over-expression and knockdown lentiviruses showed a 4.5-fold increase, and reduction of TCF21 protein levels to 8% (shTCF21 1, shTCF21 2) of baseline respectively. (TIF) [file pgen.1005155.s004.tif]

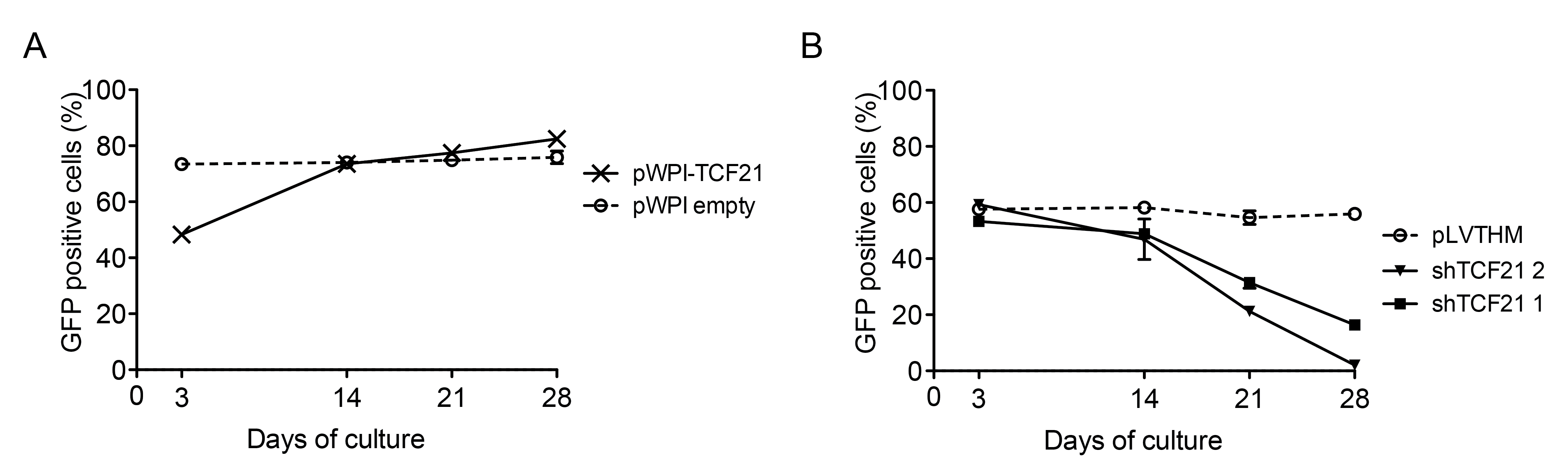

Supplement: S5 Fig — A) Flow cytometry of cultured HCASMC transduced with TCF21 overexpressing lentivirus (pWPI-TCF21) or empty lentivirus (pWPI empty), both of which express GFP, was employed to evaluate how TCF21 affects cell division. HCASMC showed an increase in TCF21 overexpressing cells from 48 to 82 percent of the culture within 25 days. B) Similar knockdown experiments were conducted with shRNA expressing lentiviruses (shTCF21 1, shTCF21 2) as well as the parent pLVTHM which served as control. All vectors expressed GFP. There was a significant decrease in GFP positive cells at day 28, siTCF21 1 vs. siCTRL P<0.0001; siTCF21 2 vs. siCTRL P<0.0001. Taken together, these data are consistent with a pro-proliferative role for TCF21. (TIF) [file pgen.1005155.s005.tif]

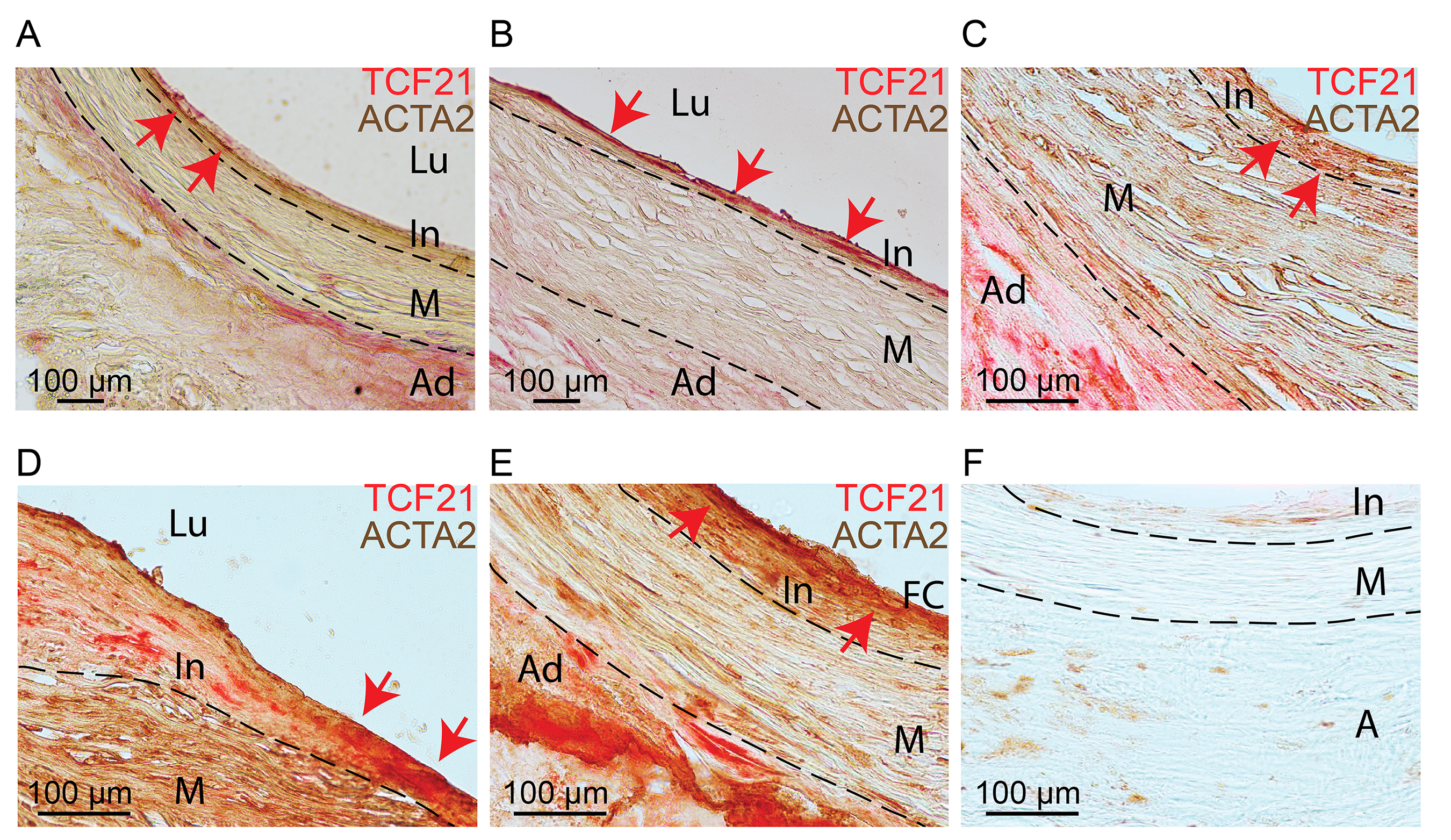

Supplement: S6 Fig — A-C) In vessels with minimal disease there was adventitial TCF21 staining (red) with some patchy staining of the minimal neointima (red arrows), but no staining in the media which was positive for ACTA2 (brown). D, E) In vessels with significant disease there was robust TCF21 staining in the adventitia and neointima and some patchy staining in the fibrous cap, but very little staining in the media. ACTA2 staining was prominent in the fibrous cap and less so in the media. F) Control studies without primary antibodies showed only weak background staining. Lu, lumen; In, neointima; M, media; FC, fibrous cap. (TIF) [file pgen.1005155.s006.tif]

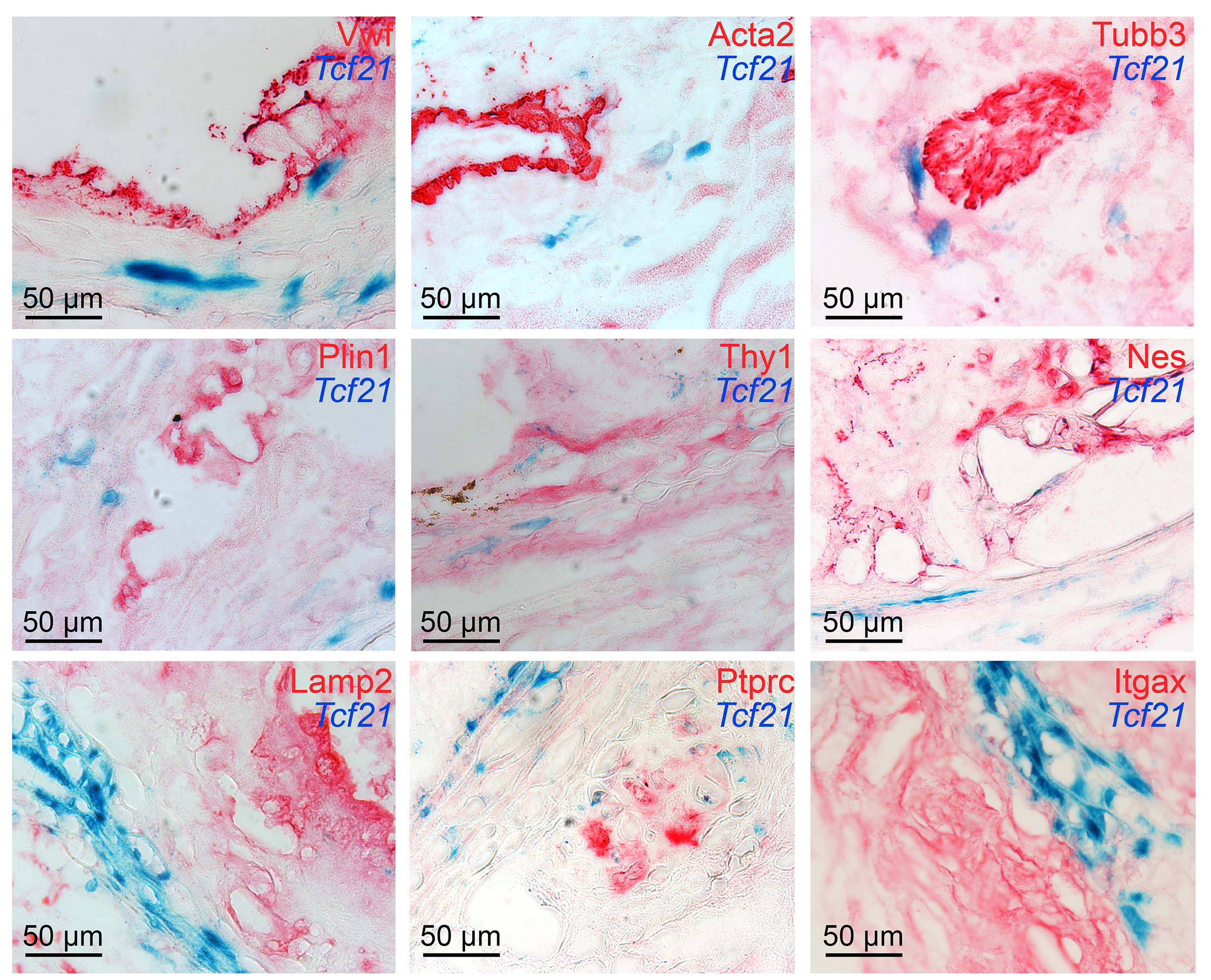

Supplement: S7 Fig — Various antibodies were employed for lineage markers with tissue from Tcf21 lacZ/+, ApoE -/- animals, Xgal stain is blue and immunohistochemical staining is red for lineage markers. (TIF) [file pgen.1005155.s007.tif]

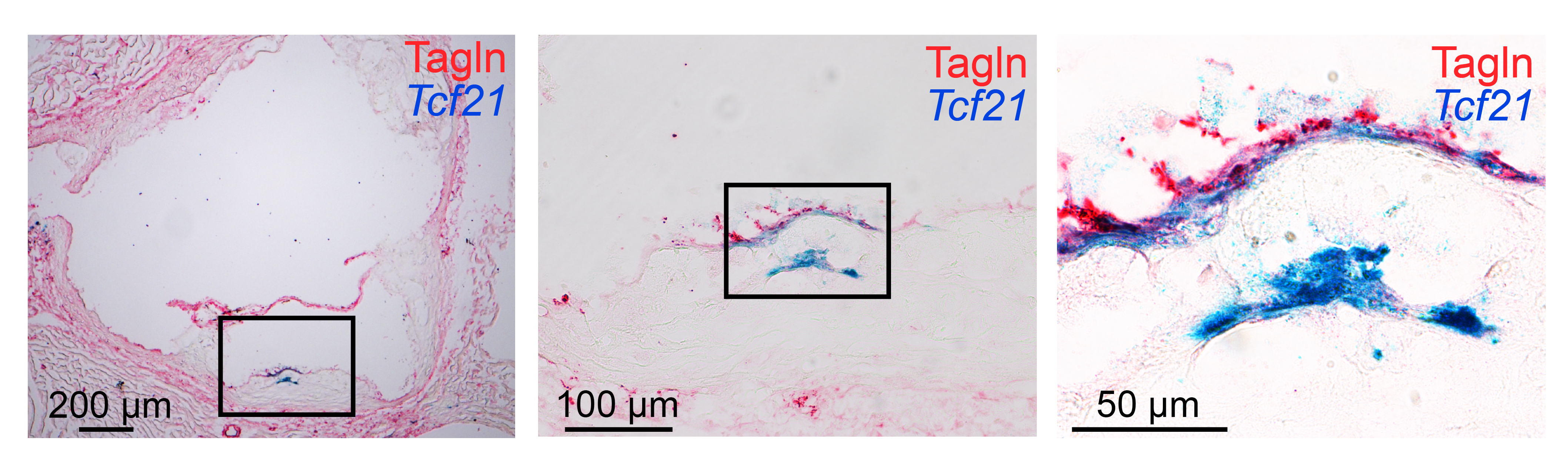

Supplement: S8 Fig — Tcf21 iCre/+, ApoE -/- mice were administered tamoxifen to activate expression of an inducible MerCreMer construct knocked into the Tcf21 locus. Cre mediated recombination of a lacZ reporter at the constitutively expressed ROSA26 locus allowed lineage tracing of Tcf21 expressing cells. Animals received tamoxifen at 6–8 weeks of HFD and tissues were harvested at 12 weeks of diet. A region of atheroma identified by the box in the low power image on the left and shown in high power fields in images on the right show blue β-galactosidase positive Tcf21 lineage traced cells forming a subcapsular structure. These cells were not labeled by immunostaining for Tagln (red). Similar β-galactosidase positive Tcf21 lineage traced cells were also seen in association with the fibrous cap and these cells did stain positive for Tagln expression. (TIF) [file pgen.1005155.s008.tif]
